# A unique profilin-actin interface is important for malaria parasite motility

**DOI:** 10.1371/journal.ppat.1006412

**Published:** 2017-05-26

**Authors:** Catherine A. Moreau, Saligram P. Bhargav, Hirdesh Kumar, Katharina A. Quadt, Henni Piirainen, Léanne Strauss, Jessica Kehrer, Martin Streichfuss, Joachim P. Spatz, Rebecca C. Wade, Inari Kursula, Friedrich Frischknecht

**Affiliations:** 1 Integrative Parasitology, Center for Infectious Diseases, Heidelberg University Medical School, Heidelberg, Germany; 2 Biocenter Oulu and Faculty of Biochemistry and Molecular Medicine, University of Oulu, Oulu, Finland; 3 Molecular and Cellular Modeling Group, Heidelberg Institute for Theoretical Studies (HITS), Heidelberg, Germany; 4 Institute for Physical Chemistry, Biophysical Chemistry, Heidelberg University, Heidelberg, Germany; 5 Department of Cellular Biophysics, Max-Planck Institute for Medical Research, Heidelberg, Germany; 6 Center for Molecular Biology (ZMBH), DKFZ-ZMBH Alliance and Interdisciplinary Center for Scientific Computing (IWR), Heidelberg University, Heidelberg, Germany; 7 Department of Biomedicine, University of Bergen, Bergen, Norway; Francis Crick Institute, UNITED KINGDOM

## Abstract

Profilin is an actin monomer binding protein that provides ATP-actin for incorporation into actin filaments. In contrast to higher eukaryotic cells with their large filamentous actin structures, apicomplexan parasites typically contain only short and highly dynamic microfilaments. In apicomplexans, profilin appears to be the main monomer-sequestering protein. Compared to classical profilins, apicomplexan profilins contain an additional arm-like β-hairpin motif, which we show here to be critically involved in actin binding. Through comparative analysis using two profilin mutants, we reveal this motif to be implicated in gliding motility of *Plasmodium berghei* sporozoites, the rapidly migrating forms of a rodent malaria parasite transmitted by mosquitoes. Force measurements on migrating sporozoites and molecular dynamics simulations indicate that the interaction between actin and profilin fine-tunes gliding motility. Our data suggest that evolutionary pressure to achieve efficient high-speed gliding has resulted in a unique profilin-actin interface in these parasites.

## Author summary

The malaria parasite *Plasmodium* has two invasive forms that migrate across different tissue barriers, the ookinete and the very rapidly migrating sporozoite. Previous work has shown that the motility of these and related parasites (e.g. *Toxoplasma gondii*) depends on a highly dynamic actin cytoskeleton and retrograde flow of surface adhesins. These unusual actin dynamics are due to the divergent structure of protozoan actins and the actions of actin-binding proteins, which can have non-canonical functions in these parasites. Profilin is one of the most important and most investigated actin-binding proteins, which binds ADP-actin and catalyzes ADP-ATP exchange to then promote actin polymerization. Parasite profilins bind monomeric actin and contain an additional domain compared to canonical profilins. Here we show that this additional domain of profilin is critical for actin binding and rapid sporozoite motility but has little impact on the slower ookinete. Sporozoites of a parasite line carrying mutations in this domain cannot translate force production and retrograde flow into optimal parasite motility. Using molecular dynamics simulations, we find that differences between mutant parasites in their capacity to migrate can be traced back to a single hydrogen bond at the actin-profilin interface.

## Introduction

Motility of adherent cells is important for both uni- and multicellular organisms. In higher eukaryotic cells, motility plays a key role in, for example embryo- and organogenesis [1], immune responses [2], and cancer metastasis [3]. Cell motility depends on an actin-myosin motor that is linked *via* transmembrane proteins to the substrate [4] (S1 Fig). In unicellular *Plasmodium* parasites that cause malaria, motility is essential for the progression through their complex life cycle [5]. The active substrate-dependent gliding motility of these parasites is essential for them to enter red blood cells, to pass through the epithelium of the mosquito midgut, and for transmission from the mosquito to the vertebrate host [5]. While the red blood cell entering merozoites are motile for just a few seconds [6], the midgut penetrating ookinetes can move for tens of minutes at approximately 5 μm/min [7–9]—almost as fast as neutrophils of the innate immune system [10]. However, the mosquito-transmitted sporozoites are the fastest form of the parasite, moving with average speeds of 1–2 μm/s for tens of minutes [11]. Sporozoites are formed in oocysts at the midgut wall and actively enter the salivary gland of the mosquito, where they can stay for weeks prior to transmission *via* a mosquito bite into the dermis of the vertebrate host [12]. In the *Plasmodium* spp. infecting mammals, the sporozoites first migrate rapidly within the dermis and subsequently enter either the blood or the lymph vessels [13]. Those entering the blood stream adhere to the liver endothelium and actively penetrate the liver parenchyma to infect hepatocytes [14].

Apicomplexan gliding motility relies on an actin-myosin motor, which resides in the narrow space delimited by the inner membrane complex (IMC), a flattened post-Golgi organelle, subtending the plasma membrane [15–17]. While myosin is anchored to the IMC, actin filaments appear to be anchored to plasma membrane traversing adhesins [18]. These provide the link between the actin filaments and the substratum [16, 17] and are essential for force transmission [19, 20]. Parasites can exert forces on various substrates in the range of 100 pN, which has been measured *in vitro* for sporozoites using traction force microscopy and optical traps [20, 21].

Efficient motility of the different stages of *Plasmodium* as well as related parasites involves unusually dynamic and short actin filaments [17, 21–24]. These remarkable properties are due to a structural divergence of the parasite actin from canonical actins [22–25] as well as a small set of regulatory actin-binding proteins, which often have non-canonical functions [26–28]. Compared to higher eukaryotic cells, there are only a few actin-binding proteins encoded in the *Plasmodium* genomes [26–29]. Gene deletion studies have shown that at least two of these proteins are not needed for the entry of merozoites into red blood cells, while they are important for the motility of ookinetes and/or sporozoites [30, 31]. Parasites lacking one subunit of the actin capping protein [31] have reduced ookinete and sporozoite motility and ultimately fail to enter the salivary glands. The actin filament binding protein coronin [32] is only expressed in *P*. *berghei* sporozoites and coronin knockout (*coronin(-))* sporozoites are not motile *in vitro* and are impaired in salivary gland invasion [30]. Curiously, *coronin(-)* sporozoites deposited into the skin by mosquito bites migrate like wild-type sporozoites [30].

Profilin, one of the core actin regulators, has been suggested to be essential in the blood stage, where transfection and selection of transgenic parasites is performed [33]. Indeed, conditional down-regulation of profilin, likely leading to a defect in merozoite invasion of red blood cells, suggested that it is important for efficient blood stage growth [34], while overexpression had no effect on the blood stage [35]. Canonical profilins bind monomeric ADP-actin, catalyze ADP-to-ATP exchange, and enhance actin polymerization in conjunction with the actin nucleators of the formin family [36]. Importantly, apicomplexan formins contain only rudimentary or no profilin-binding motifs, and both *P*. *falciparum* and *T*. *gondii* profilins appear to sequester actin monomers also in the presence of formins [37, 38]. Furthermore, *T*. *gondii* profilin inhibits, rather than accelerates, nucleotide exchange on actin monomers [38, 39]. Thus, it was suggested that *T*. *gondii* profilin functions mainly as an actin monomer sequestering protein, possibly limiting actin polymerization *in vivo* [38].

Profilin may have evolved *via* gene fusion from two smaller proteins [40]. Subsequent divergent evolution seems to have resulted in two structurally very different branches: the classical profilins in most organisms as well as the apicomplexan ones with a large sequence insertion at the border of the two small ancestral proteins [33, 40]. This insertion, a unique arm-like ß-hairpin domain seems optimally positioned to participate in actin binding [33, 39]. However, a possible functional contribution of this motif in both actin binding and parasite motility remained uncharacterized so far. To probe this potential interaction and to investigate the structure-function relationships of profilin across the *Plasmodium* life cycle, we generated a series of transgenic parasites, featuring two mutations at the tip of the ß-hairpin. We further expressed the corresponding proteins *in vitro* and show that the arm-like domain contributes critically to the actin-binding capacity of *Plasmodium* profilin. Interestingly, this additional motif appears important only during the rapid motility of sporozoites, where a weakened profilin-actin interaction leads to a decrease in their capacity to generate force. Strikingly, one mutant still retained continuous gliding motility, while the other one lost this ability, although for both mutants, the capacity to produce force was similarly weak. Molecular dynamics simulations suggested differences in the actin-binding mode of the two mutants, which appears to be at the core of the different gliding phenotypes. Taken together, our data show how sporozoites fine-tune actin dynamics to achieve gliding motility.

## Results

### Profilin is expressed during the entire *Plasmodium* life cycle and localized throughout the parasite

**Fig 1:**
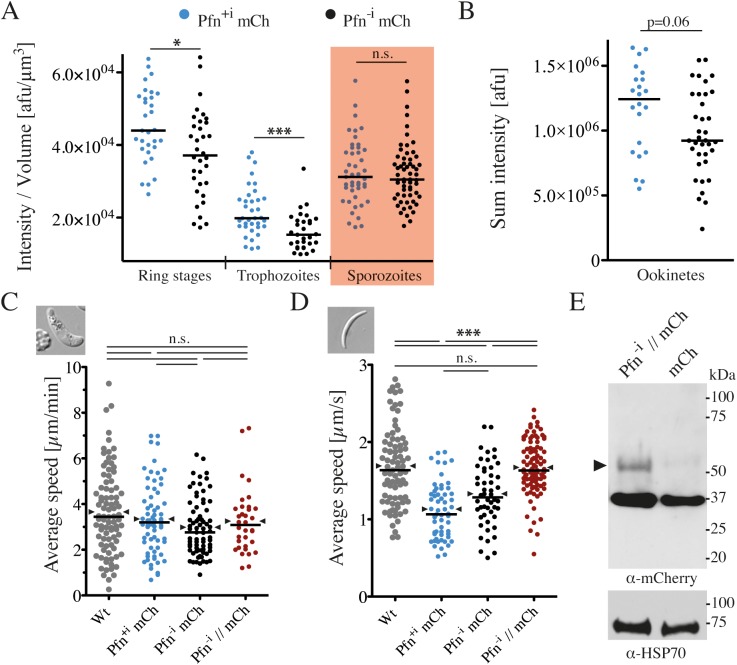
Fluorescent tagging and absence of introns can impact profilin function. (A) Quantification of profilin-mCherry fluorescence per volume in ring stages, trophozoites and sporozoites (red shading). Each dot represents the fluorescence intensity normalized to parasite volume for one parasite. Horizontal bar: median. Significances were tested with Mann-Whitney tests; * denotes p<0.05; *** p<0.001.(B) Quantification of total profilin-mCherry fluorescence intensity of ookinetes. Note that fluorescence was much stronger in the nucleus of ookinetes (S3B Fig) and thus ookinetes were not directly comparable with other stages. Significance was tested with a Mann-Whitney test. (C, D) Average speed with median (line) and mean (arrowheads) of ookinetes (C) and sporozoites (D) from the different parasite lines; Wt: wild type; Pfn^+i^ mCh: Profilin-mCherry with introns; Pfn^-i^ mCh: Profilin-mCherry without introns; Pfn^-i^ // mCh: Profilin-mCherry without introns and long linker, which is cleaved (see panel E). At least 50 parasites per line were tracked over at least half of the acquisition time (sporozoites: 5 min, ookinetes: 15 min); significance was tested with a Kruskal-Wallis test; *** denotes p<0.001. (E) Western blot showing a weak band for the profilin-mCherry fusion protein (arrowhead) and a major band for a cleaved mCherry of the parasite line expressing profilin-mCherry with a long linker (Pfn^-i^ // mCh) detected with an anti-mCherry antibody in blood stage schizont lysates (lane 1). Note that at least 75% of profilin-mCherry is cleaved. Lane 2: control line expressing only mCherry. Loading control: anti-HSP70.

*P*. *berghei* profilin has previously been shown by RT-PCR analysis to be expressed across the entire parasite life cycle [33]. To investigate profilin protein abundance and localization across the life cycle, we endogenously tagged profilin with mCherry (S2 Fig). Since the profilin gene consists of 4 exons and 3 introns, we measured expression of profilin-mCherry constructs with and without introns (Pfn^-i^ mCh and Pfn^+i^ mCh), as these could influence gene expression [41, 42]. The fluorescent parasites progressed through the life cycle in a manner largely comparable to wild-type parasites, suggesting that the mCherry fusion did not detectably impair parasite viability (Table 1). Western blot analysis showed that profilin-mCherry migrated at the expected size of the fusion protein (S3A Fig). Imaging parasites at the ring, trophozoite, and sporozoite stages revealed uniform profilin localization in the cytosol and the nucleus, while in gametocytes and ookinetes the profilin-mCherry fluorescence was more pronounced in the nucleus (S3B Fig). Profilin localization is consistent with the localization of GFP-actin, which was also found in the cytosol and the nucleus [43]. Addition of actin filament stabilizing (jasplakinolide) or depolymerizing (cytochalasin D) compounds did not alter the localization of profilin, as expected for an actin monomer binding protein. Quantitative analysis of images from different stages showed that the profilin-mCherry expression levels varied slightly across the stages. The only significant differences in expression depending on the presence or absence of introns occurred in the blood stages (Fig 1A). Intriguingly, the parasites lacking introns in the profilin gene grew slightly more slowly in the blood stage (Table 1) and reached slightly larger cell sizes of up to 45 μm^3^ (S3C and S3D Fig). The fluorescence intensity of Pfn^+i^ mCh and Pfn^-i^ mCh motile ookinetes also appeared slightly different (p = 0.06), yet the Pfn expression in sporozoites was not affected (Fig 1A and 1B). Curiously, while the speed of motile ookinetes was not affected (Fig 1C), the speed of sporozoites expressing mCherry-tagged profilin both with and without introns was significantly reduced (Fig 1D). This suggests steric hindrance by the mCherry tag that only has an effect on motile sporozoites. The speed of sporozoites could be restored to wild type levels in parasites that expressed a longer linker between profilin and mCherry (Pfn // mCh) (Fig 1D). However, in these parasites, the mCherry tag was largely cleaved off, leading to a majority of non-tagged profilin (Fig 1E). In all tagged lines, Pfn expression was under the control of the endogenous promoter and the 3’UTR from *Pb*DHFR, suggesting that the 3’UTR did not influence the level of profilin expression (S2A Fig). These data highlight the importance of carefully evaluating the impact of large fluorescent tags on protein functionality.

**Table 1:** Comparative life cycle progression of the different parasite lines.

Parasite line	Blood-stage growth rate	Oocysts / infected MG	MG spz / mosquito	SG spz / mosquito	Prepatency [days]	Parasitemia d6 [%]
*Pb* WT	8.9	105	39 000	9 400	3.75	2.3 ± 0.4
Pfn^+i^ mCh	9.2	90	38 000	7 600	3.5	1.7 ± 0.6
Pfn^-i^ mCh	8.5	75	37 000	6 400	4	1.4 ± 0.1
*Pf* Pfn	10.5	75	36 000	4 300	4	1.7 ± 0.8
QNQ Pfn	9.2	80	37 000	7 400	4.5	1.7 ± 0.7
AAA Pfn	9.5	90	50 000	7 300	4.25	1.5 ± 0.3
	n≥8	n≥50	n≥3	n≥3	n = 4	n = 4

Analysis of parasite lines expressing mCherry-tagged profilin with or without introns or different variants of *P*. *falciparum* profilin. Median blood-stage multiplication rates were determined after injecting either 100 or 5000 blood stages intravenously into four C57BL/6 mice respectively. Oocyst numbers (mean) were obtained by counting the cysts of at least 50 infected mosquito midguts (MG) on day 12. MG and salivary gland (SG) sporozoite (spz) numbers (mean) were counted between days 17 and 25. For each parasite line, at least three different cages were assessed, and the resulting mean is displayed. Parasitemia on day 6 and prepatency (first day of detectable blood-stage infection) were determined for each line after four C57BL/6 mice were bitten by 10 mosquitoes.

### *P*. *falciparum* profilin can functionally complement *P*. *berghei* profilin

As human profilin has previously been shown to partially compensate for *P*. *berghei* profilin depletion [33], we hypothesized that *P*. *falciparum* profilin should also compensate for *P*. *berghei* profilin depletion. We reasoned that this would enable detailed mutational analysis in a more straightforward fashion (see methods) and with the protein of a disease-relevant parasite species that has already been expressed and analyzed *in vitro* [33]. Considering also the lack of striking differences in expression in the presence or absence of introns, we thus generated a replacement plasmid using *P*. *falciparum* cDNA and obtained a parasite line that showed no difference to wild type *P*. *berghei* parasites across the life cycle (S2 Fig, Table 1), suggesting that the two profilin orthologues are functionally interchangeable.

### Molecular dynamics simulations suggest involvement of the *Plasmodium*-specific motif in the profilin-actin interaction

**Fig 2:**
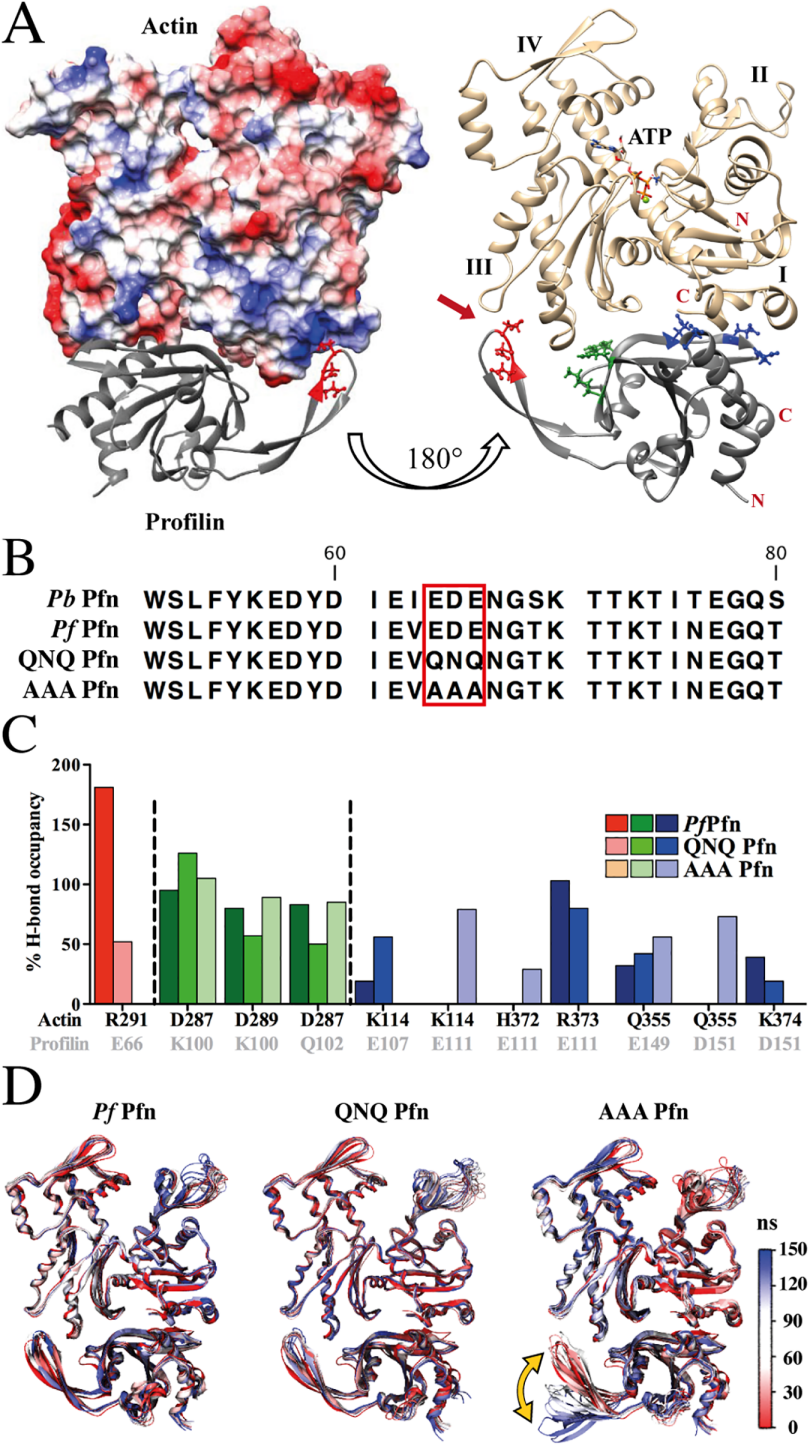
The arm motif of *Plasmodium* profilin is critical for actin binding. (A) Models of the *P*. *falciparum* profilin-actin 1 complex from molecular dynamics simulations. Note the tip of the arm-like β-hairpin (acidic residues in red) contacting a basic region of actin subdomain 3. Left: The surface of actin is colored by electrostatic potential from blue (basic) to red (acidic). The right panel shows actin in gold and profilin in grey; the actin-profilin complex is shown with different contact residues highlighted in red (tip of hairpin), green (arm-neighbor interactions) and blue (classic actin-profilin interface encompassing β-strands 4 and 5). Subdomains 1–4 are labelled in Roman numerals and the Apicomplexa-specific actin-profilin interface is indicated with a red arrow on the right. (B) Amino acid sequence of the arm domain from *P*. *berghei* and *P*. *falciparum* profilins showing the AAA and QNQ mutations. (C) Analysis of the hydrogen bonds during 100–150 ns of molecular dynamics simulation formed by the residues indicated with the matching colors in A. The graph shows the occupancy of hydrogen bonds between the indicated amino acid residue pairs for the *Pf* Pfn and the QNQ and AAA mutants. Note that the AAA mutant shows no interaction at the tip of the hairpin. K100, Q102, E107 and E111 were previously predicted to interact with actin [33]. Values higher than 100% occupancy are due to formation of more than one hydrogen bond. (D) Ensemble structures of *Plasmodium* profilin in complex with *Plasmodium* actin during 150 ns of molecular dynamics simulations. All trajectories are aligned to the backbone of the WT complex (frame 1). Red highlights the starting and blue the final structure. Note the large mobility of the arm-like hairpin in the AAA mutant (yellow double-arrow).

The structure of *Pf* Pfn suggested that the parasite-specific hairpin and in particular the acidic residues at the tip of this loop might be involved in interactions with actin, presenting a unique actin-profilin interface [33]. The profilin-binding surface in *Pf* actin 1 is conserved with canonical actins. However, the actin-binding surface in *Pf* Pfn is remarkably diverged. To test whether *Pf* profilin binds to actin at the canonical interface, we expressed *Pf* profilin in *E*.*coli* and purified the protein (see methods). Incubation at a 1:1 ratio with pig muscle actin allowed investigation of complex formation by small-angle X-ray scattering. We found that the shape of the complex corresponds to that of canonical profilin-actin complexes (S4 Fig). Furthermore, increasing concentrations of *Pf* Pfn increase the fluorescence signal of the pyrene-labelled Cys located in the C-terminal helix of actin at the canonical profilin binding site (S5A and S5B Fig), indicating that *Pf* Pfn binds to the canonical binding site on actin. To investigate the parasite actin-profilin interface in more detail, we constructed a homology model of the *Pf* Pfn-actin complex and used it for molecular dynamics simulations. *P*. *berghei* actin and *P*. *falciparum* actin share 99.2% sequence identity, differing at only three residues: E3D, D5E and V11I, meaning that their electrostatic properties at the profilin binding face are very similar. We first analyzed the electrostatic properties of the modelled interface between *Plasmodium* actin and *P*. *falciparum* profilin (*Pf* Pfn). The complex can be superimposed on the rabbit actin-human profilin complex (PDB ID: 2PAV) [44], but there are remarkably large differences in the two profilin-actin complexes. In addition to the arm-like β-hairpin insertion in *Pf* Pfn (Fig 2A), the lack of the short α-helix 3 and the much longer β-strands 4 and 5 in *Pf* Pfn cause significant differences at the interface. As hypothesized before, the acidic residues _64_EDE_66_, conserved across *Plasmodium* spp., at the tip of the parasite-specific arm (Fig 2B) are in close contact with a positively charged surface patch on subdomain 3 of actin in our model, suggesting the possibility to form a strong ionic (salt-bridge) interaction (Fig 2A, S1 Movie). The actin residues contributing to this positive patch include K284, R291, K292, Y295 and T325 (numbering as *per Pf* actin 1) and are conserved between *Plasmodium* and vertebrate skeletal muscle α-actin, suggesting that *Pf* Pfn can engage in similar interactions with both actins.

To get more insight into how the acidic residues of the arm motif in *Pf* Pfn contribute to actin binding, we simulated actin-profilin complexes with two mutant versions, in which the residues _64_EDE_66_ were exchanged to QNQ or AAA (Fig 2B, S2 Movie). These mutations were chosen in order to weaken (QNQ) or abolish (AAA) the interactions of the acidic arm motif with actin. We analyzed the inter-protein hydrogen-bond occupancy during simulations for three interaction regions of the protein-protein interfaces: the arm-specific interaction (shown in red in Fig 2A and 2C); arm-neighbor interactions (shown in green in Fig 2A right panel, C); and the conventional actin-profilin interface interactions (shown in blue in Fig 2A right panel, C). At the tip of the arm motif, E66 of *Pf* Pfn makes two strong hydrogen bonds with R291 of actin with the highest occupancy (181% out of a possible 200% occupancy for 2 hydrogen bonds, individually 90% and 91%) of all the hydrogen bonds at the actin-profilin interface (Fig 2C). In the QNQ mutant, Q66 lacks the negative charge and therefore loses the salt-bridge nature of this interaction (S1 Movie). Nevertheless, Q66 of the QNQ mutant profilin can form a single hydrogen bond with actin-R291 (52% occupancy) (Fig 2C). On the other hand, the AAA mutant as predicted is unable to make any arm-mediated ionic or hydrogen-bonding interactions at the actin-profilin interface (Fig 2C and 2D, S2 Movie).

In contrast to the profilin arm-mediated interactions, the hydrogen bonds in the vicinity of the Pfn arm are preserved in the two mutants. Surprisingly, the hydrogen-bonding residue-pairs significantly differ at the interface formed by β-strand 4 of *Pf* Pfn (Fig 2C), and the AAA mutant has the most divergent hydrogen-bonding pattern in this region. For example, while E111 of *Pf* Pfn makes strong hydrogen bonds (103%) with actin R373, these have lower occupancy (80%) in the QNQ mutant and are absent in the AAA mutant. However, in the AAA mutant, E111 forms hydrogen bonds with K114 (79%) and H372 (29%) in actin. The loss of these hydrogen bonds in the QNQ mutant is compensated by an additional stronger hydrogen bond: E107 with actin-K114 (56%) (S6 Fig).

The weakening of the interaction with actin for the AAA mutant, due to the lacking hydrogen bonds involving the tip of the β-hairpin loop, is also visualized by molecular dynamics simulations, during which the arm region in the AAA mutant is destabilized and moves away from actin (Fig 2D, S2 Movie). This destabilization further weakens the interactions at the conventional actin-profilin interface involving β-strand 4 (Fig 2C, blue bars).

We finally computed the relative free energy values (lacking the full entropic contribution) for the wild type and mutant *Pf* Pfn-actin complexes (Table 2). This suggests that wild type *Pf* Pfn binds most strongly to actin (MM-PBSA free energy: -64.2 ± 11.2 kcal/mol; similar values and the same trends were obtained with the MM-GBSA method). The QNQ mutant has a less favorable binding free energy (-44.2 ± 12.8 kcal/mol), which is in line with its weaker H-bond interactions. The AAA mutant is the least energetically stable (-34.4 ± 11.1 kcal/mol) of the complexes, which may be due to the instability of the arm region which further destabilizes other interactions at the actin-profilin interface.

**Table 2:** MM-PBSA and MM-GBSA estimates of binding free energies of wild type and different mutant complexes.

	*Pf* Pfn		QNQ Pfn		AAA Pfn	
	Avg	S.D.	Avg	S.D.	Avg	S.D.
**MM-PBSA**	-64.2	11.2	-44.2	12.8	-34.4	11.1
**MM-GBSA**	-61.8	9.2	-50.7	10.8	-45.6	8.3

Average (Avg) binding energies were calculated from the last ns (50 frames) of the simulation and are displayed with standard deviations (S.D.). The unfavorable entropy energies were not calculated. All values are in kcal/mol.

### Mutations in the *Plasmodium*-specific arm domain of profilin lead to impaired actin binding

**Fig 3:**
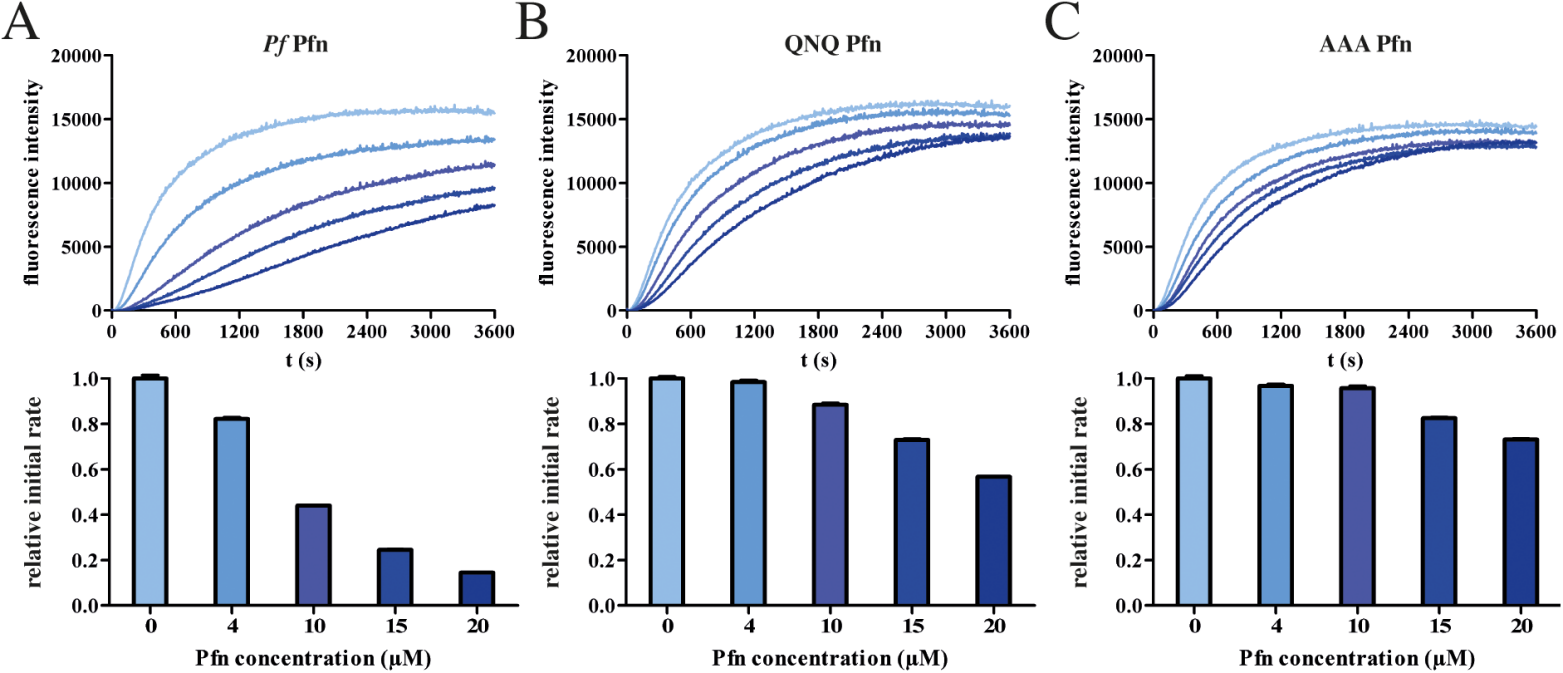
Effect of *Pf* and mutant profilins on pig muscle α-actin polymerization. Polymerization curves and relative initial polymerization rates of 4 μM α-actin alone and in the presence of 4–20 μM *Pf* wild type (A), QNQ (B) and AAA (C) profilins. Initial polymerization rates are depicted below the curves and were determined for the time frame of 300–600 s.

**Fig 4:**
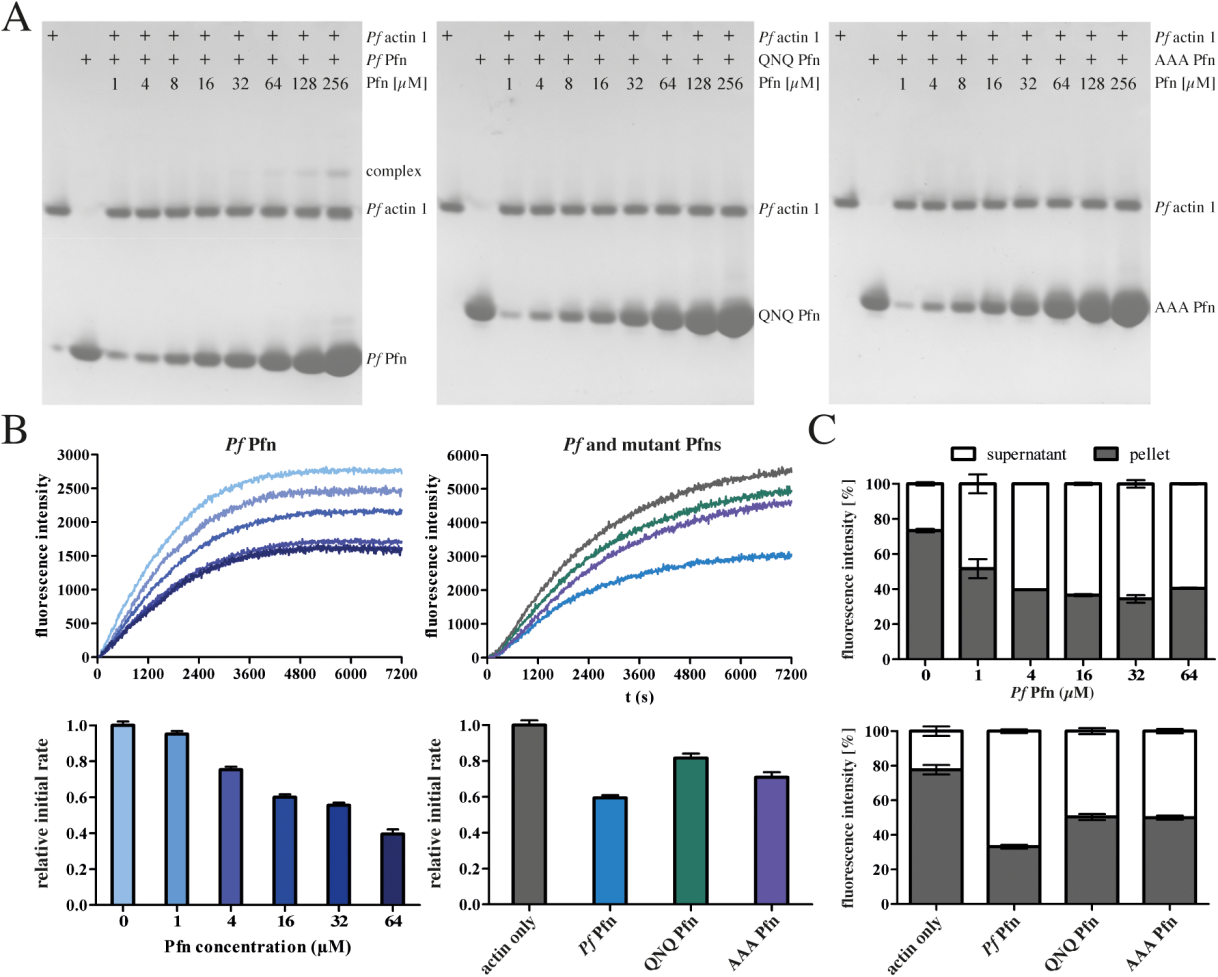
Effect of *Pf* and mutant profilins on *Plasmodium* actin. (A) Native-PAGE analysis of wild type and mutant *Pf* Pfn binding to *Pf* actin 1. Note the absence of complex formation in the QNQ and AAA mutants. (B) Polymerization and relative initial polymerization rates of 4 μM *Pf* actin 1 alone and in the presence of 1–64 μM *Pf* profilin (left panels) and 16 μM *Pf*, QNQ and AAA profilins (right panels). Initial polymerization rates are depicted below the curves and were determined for the timeframe of 600–1200 s. (C) Quantification of co-sedimentation analysis of *Pf* actin 1 with 16 µM wild-type (upper panel) and mutant (lower panel) *Pf* Pfns from duplicate samples. Representative gels are presented in S7C Fig.

To investigate whether the differences suggested by the simulations can lead to biochemically detectable differences in actin binding of the mutants, we next characterized the effect of recombinant *P*. *falciparum* profilin and both mutants on the polymerization kinetics of vertebrate skeletal muscle α-actin. Like wild-type *Pf* Pfn, both QNQ and AAA mutants could be purified as monomeric, soluble, and folded proteins **(**S7A and S7B Fig**)**. Whereas wild-type *Pf* Pfn drastically reduced the rate of canonical actin polymerization, both mutants showed a significantly reduced effect (Fig 3). The weaker binding of the mutants to actin was also seen in the lack of increased initial fluorescence (S5C and S5D Fig). We next assessed, whether the same would be true for the biologically relevant binding partner, *Pf* actin 1. Using native PAGE, complex formation was observed for wild type but not for the mutant profilins (Fig 4A). However, intriguingly, the effects of *Pf* Pfn on canonical and parasite actin polymerization kinetics were different. While *Pf* Pfn significantly reduced the rate of elongation for both actins, it had little effect on the steady-state of α-actin (Fig 3A), but a clear monomer-sequestering activity on *P*. *falciparum* actin 1 (Fig 4A and 4B). The mutated profilins showed a weakened capacity for reducing the rate of polymerization as well as monomer sequestering (Figs 3 and 4). However, there was no clear difference in the effects of the QNQ and AAA mutants, especially on parasite actin **(**Fig 4**)**, suggesting that even the relatively subtle differences in the binding of the QNQ mutant compared to the wild type *Pf* Pfn are sufficient for significantly reducing the binding affinity to actin.

### Parasites with mutations in the *Plasmodium*-specific arm domain of profilin show aberrant sporozoite motility

**Fig 5:**
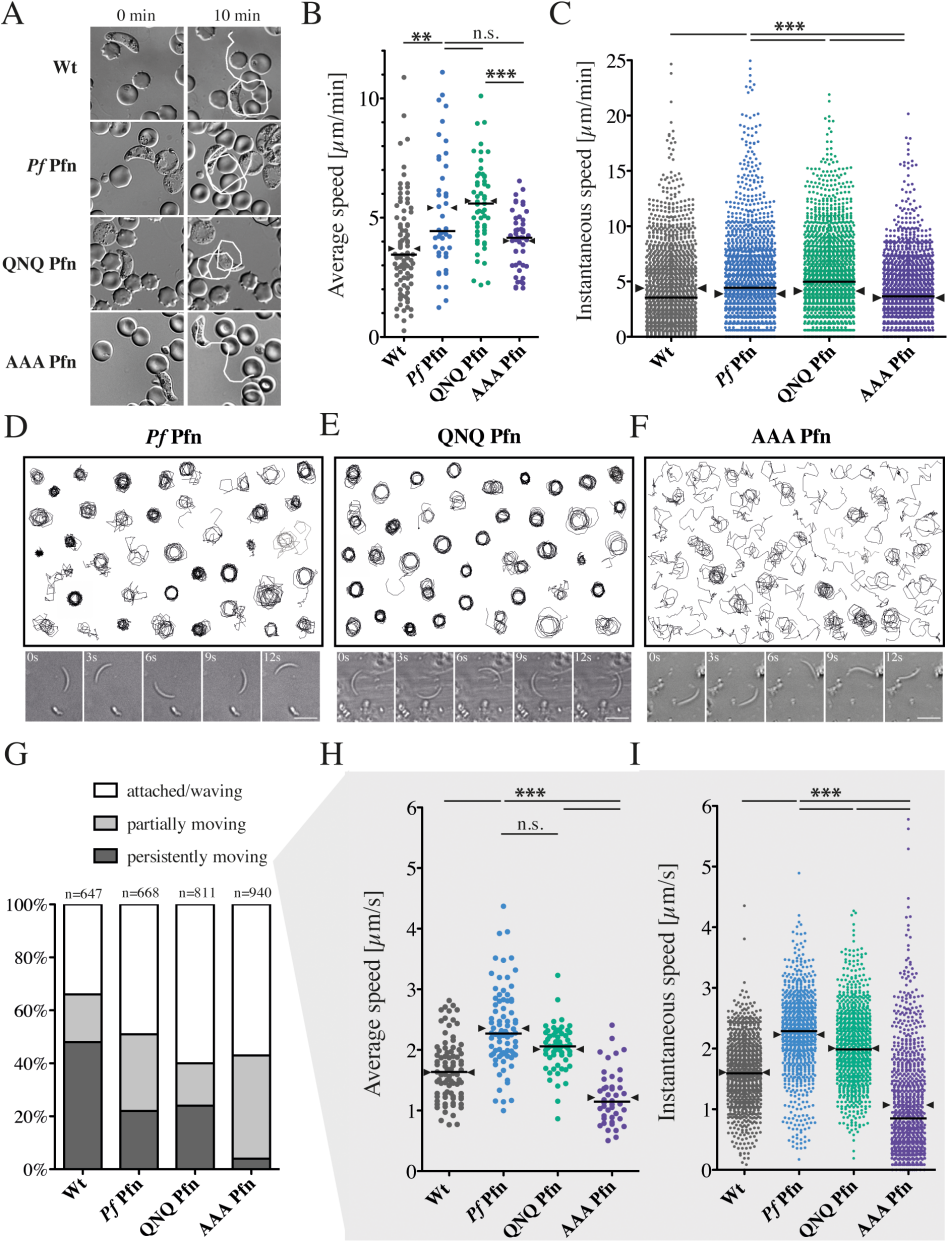
Aberrant motility of *P*. *berghei* sporozoites expressing *P*. *falciparum* ‘AAA profilin’. (A) Representative images of ookinetes from the indicated lines moving for 10 minutes. White lines indicate tracks after 10 min. (B) Average speeds of ookinetes with median (line) and mean (arrowheads) from different parasite lines. At least 40 parasites per line were tracked over 15 min; significance was tested with a one-way ANOVA; ** denotes p<0.01, *** p<0.001. Horizontal bar: median, arrowheads indicate mean. (C) 10 ookinetes with median average speeds were plotted for their instantaneous speeds (time lapse: 20 s); significance was tested with a Kruskal-Wallis test; *** denotes p<0.001. Horizontal bars: median, arrowheads: mean. (D-F) Assembly of 40 randomly selected sporozoite tracks with representative time-lapse images below expressing (D) wild type, (E) QNQ and (F) AAA *P*. *falciparum* profilin. Images below are 3 seconds apart. Scale bar: 10 μm. Note the different patterns of migration in the AAA mutant. (G) Movement patterns of sporozoites clustered within three groups: ‘persistently moving’ denotes parasites that showed gliding motility for at least 50 consecutive frames (recorded at 0.33 Hz) without their speed dropping below 0.2 μm/s for more then 10 frames; ‘partially moving‘ denotes parasites that showed some circular gliding motility but did not fulfill the ‘persistently moving‘ criteria and ‘attached/waving‘ [74] denotes parasites that did not display any active circular gliding. (H) Average speeds of persistently moving sporozoites tracked between 50 and 100 frames (150–300 s) for at least 50 sporozoites, *** denotes p<0.001; Kruskal-Wallis test. Horizontal bars: median, arrowheads: mean. (I) 10 sporozoites with median average speeds were plotted for their instantaneous speeds (from one frame to the next = 3 s). Note the shift of AAA sporozoites to a high abundance of very slow speeds as well as some notably high speeds, *** denotes p<0.001; Kruskal-Wallis test. Horizontal bars: median, arrowheads: mean.

To address whether the mutations would lead to a detectable effect *in vivo*, we generated parasites expressing the respective *P*. *falciparum* profilins in place of the single endogenous *P*. *berghei* profilin gene (S2 Fig) and compared them to wild type *P*. *berghei* parasites. Expression of the QNQ and AAA mutants in the parasite had no detrimental effect on life cycle progression (Table 1). Whereas only minor differences in the speed of wild type and transgenic ookinetes were observed (Fig 5A–5C), a striking effect was seen in sporozoites (Fig 5D–5I). We found no difference between the wild type and QNQ sporozoites in their capacity to glide on a flat substrate, but the AAA mutants largely failed to move progressively (Fig 5D–5G). Curiously, *Pf* Pfn-expressing sporozoites moved faster but less persistently than wild type *P*. *berghei* sporozoites, and this was also seen for the QNQ mutant (Fig 5G and 5H). The differences in speed were even more obvious when instantaneous speeds were compared (Fig 5I). Interestingly, also the AAA mutant ookinetes moved at lower speeds than the QNQ mutant ookinetes (Fig 5B). Together with the biochemical data and the molecular dynamics simulations, these data on sporozoite motility confirm that the parasite-specific arm in *Pf* Pfn is crucial for actin binding and, thereby, sporozoite motility. It is interesting that both simulations and motility assays show differences between the QNQ and AAA mutants, but these are not obvious from the biochemical assays, suggesting that the subtle differences in the two mutants are more complicated than a simple effect on binding affinity.

### Sporozoites expressing profilin mutants produce lower forces

**Fig 6:**
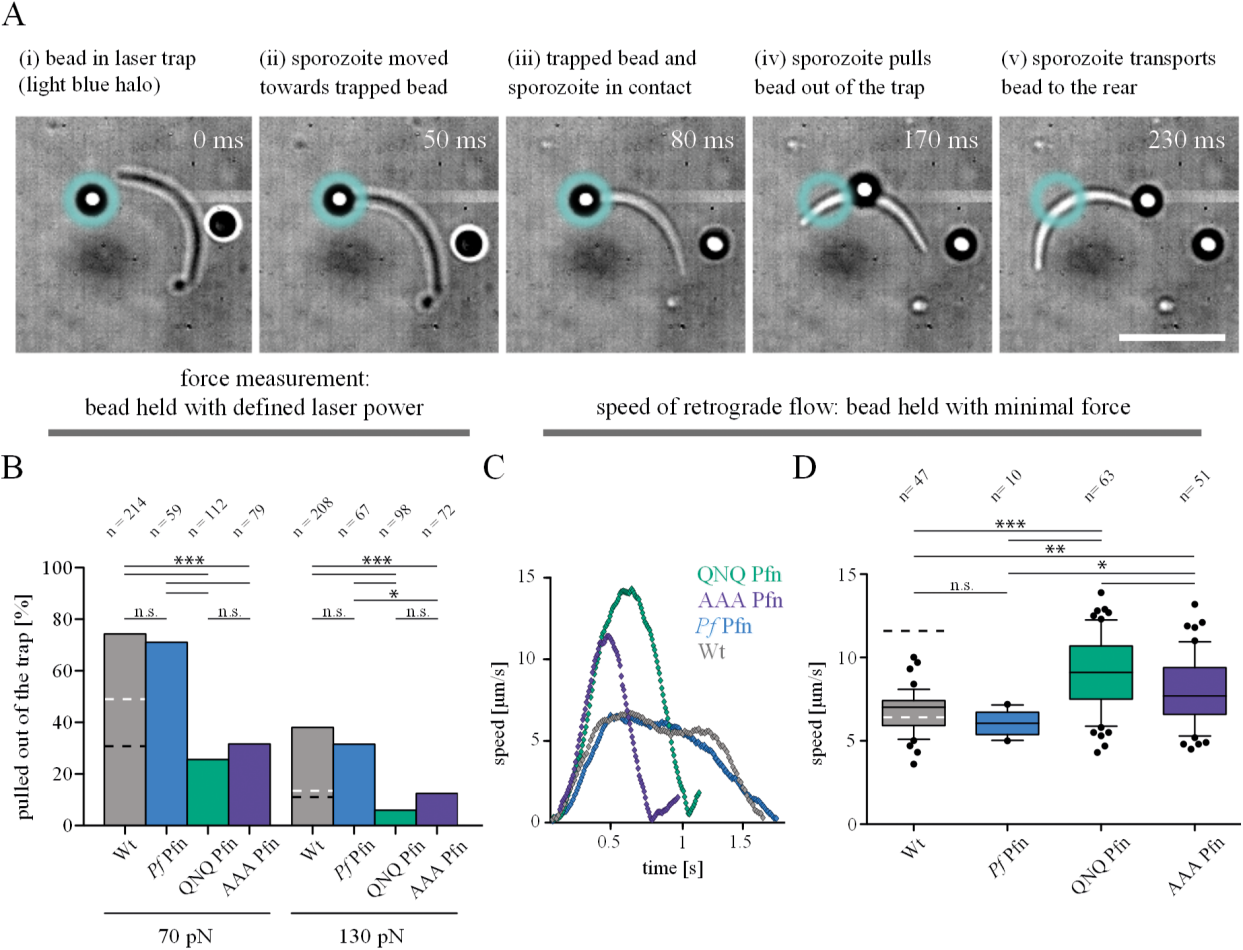
Sporozoites expressing profilin mutants generate less force but faster retrograde flow rates. (A) Image series of a sporozoite pulling a polystyrene bead out of a laser trap. (i-iii) The bead is held in the trap and put in contact with the sporozoite surface; (iv) the sporozoite pulls the bead out of the trap; (v) the sporozoite transports the bead to the rear end. Scale bar: 10 μm. (B) Quantitative analysis of the capacity of *P*. *falciparum* expressing sporozoites to pull a bead out of the trap from > 200 sporozoites tested with two to three different counter forces over 2–3 separate days. Bars represent the percentage of sporozoites that managed to pull the bead out of the trap. Black and white dashed lines indicate the level of wild type sporozoites treated with 50 nM jasplakinolide or cytochalasin D, respectively as published in Quadt et al., 2016 [20]. Significance was determined by Fishers exact test. (C) Representative speed plots of four beads tracked on four different sporozoites expressing either wild type *P*. *berghei* or *P*. *falciparum* profilin or mutated versions of the latter (namely QNQ or AAA). (D) Retrograde flow speeds as measured from over 170 sporozoites of the indicated parasite lines; boxes represent 50% of data, horizontal bar: mean, whiskers with 10–90 percentiles. Black and white dashed lines indicate the percentage of wild type sporozoites treated with 50 nM jasplakinolide or cytochalasin D, respectively that managed to pull a bead out of the trap as published in Quadt et al., 2016 [20]. Significance was determined by unpaired t-test. Note the higher peak values in the mutants of up to 14 μm/s compared to around 7 μm/s for wild type sporozoites.

Mutations of the unique profilin arm clearly emphasize the importance of this region in facilitating efficient sporozoite motility. However, the phenotypic characterization described so far, does not yet lead to a functional understanding of how profilin-actin interactions modulate parasite motility. To gain further functional understanding, we employed a recently-established laser trap assay [20]. This allowed us to probe at defined forces the capacity of sporozoites to pull a micron-sized bead from an optical trap and to translocate it to the rear of the cell (Fig 6). We presume that this pulling force corresponds to the force that sporozoites exert on their surrounding environment to move forward. To measure this force, a bead was trapped in the laser focus and pushed onto the sporozoite surface, where it was captured by the sporozoite (Fig 6A). Subsequently, the sporozoite pulled the bead backwards, presumably due to the myosin-based translocation of actin filaments anchored *via* transmembrane adhesins [20]. This translocation backwards thus most likely corresponds to the retrograde flow of actin filaments observed in fibroblasts and other cells and also assumed to occur in sporozoites [20, 30, 45]. We probed our set of parasites with this assay and scored whether, at a given optical force, the sporozoite pulled the bead out of the trap or not. In total, over 1,200 sporozoites were probed from 4 different parasite lines at 3 different optical forces (Fig 6B–6D). In this very sensitive assay, no difference was found between wild type *P*. *berghei* parasites and *P*. *berghei* parasites expressing *P*. *falciparum* profilin. At an optical force of 70 pN, sporozoites from both lines were able to pull approximately 70% of the beads out of the trap, while at 130 pN, this percentage dropped to approximately 35% (Fig 6B, [20]) and at 190 pN to 20% [20]. Both the AAA and the QNQ mutant were less efficient in pulling the bead from the trap at all forces (Fig 6B). At 70 pN, both mutant lines only managed to pull approximately 20% of beads out of the trap, and at 130 pN, this dropped further to approximately 10%.

### Profilin mutants show enhanced retrograde flow rates

To probe whether the mutations affect retrograde flow, we used the same laser trap setup. However, in these experiments the force of the trap was set to approximately 10 pN, which was sufficiently small for the sporozoite to instantly move the bead towards the rear (Fig 6A). In all parasite lines, the beads were transported backwards with an increasing speed until a peak or plateau was reached (Fig 6C). To analyze potential differences in the transport speeds, we compared the maximum speed of over 170 sporozoites across the four different parasite lines. We found no difference between the wild-type *P*. *berghei* and the line expressing wild type *P*. *falciparum* profilin (Fig 6C and 6D). However, parasites expressing the AAA and QNQ mutant profilins moved the beads at an increased speed, *i*.*e*. showed a faster retrograde flow (Fig 6C and 6D). Thus, while the QNQ mutant seems impaired in actin binding and force generation to a similar degree as the AAA mutant, there are subtle differences that lead to differences in the ability of the mutants to support fast, directional motility. The molecular dynamics simulations suggest this could be due to differences in the off rates of the complexes, especially in the situation *in vivo*, where other proteins compete with profilin for actin binding.

## Discussion

### A unique actin-profilin interface important for motility

Profilins as ancient actin-binding proteins have typically low sequence identity but share a highly conserved 3D fold. Apicomplexan profilins differ from all other profilins in containing a unique β-hairpin extension. The exact length and sequence of this insertion is not fully conserved between different *Apicomplexa*, but they all share the acidic nature of the tip of this extension. Here, we show that mutations in this unique motif in *Plasmodium* diminished actin monomer sequestration *in vitro* (Figs 3 and 4) and led to impaired sporozoite motility (Fig 5G–5I), while only slightly affecting ookinete motility (Fig 5B and 5C). This differential effect was already visible in the construction of profilin-mCherry fusions, which slightly impaired sporozoite motility while they had no effect on ookinete motility (Fig 1C and 1D). The defects were more accentuated in the mutant parasite lines analyzed. This, together with previous data [31] suggests that for the evolution of the core motility machinery, the sporozoite presents the major constraining stage of the parasite, as it appears the most vulnerable to subtle changes. This is likely due to the fact that the sporozoite has to migrate extremely fast, while passing several biologically very different tissue barriers.

### Mutants reveal a stepwise loss of actin-profilin interaction

Our data revealed an interesting dichotomy: while both QNQ and AAA mutants showed similar differences compared to wild type in our biochemical and biophysical assays, only the AAA mutant showed a defect in motility. The molecular dynamics simulations might provide an answer for this intriguing difference. They revealed a salt bridge with two hydrogen bonds that contributes to stabilizing the interaction between the arm-motif of profilin and actin (Fig 2, S1 Movie). Interestingly the salt bridge is lost in both mutants, whereas one of the two hydrogen bonds is retained in the QNQ mutant but both hydrogen bonds are lost in the AAA mutant. This suggests that the biochemical and biophysical assays were sensitive to the differences due to the loss of the salt bridge and one of the hydrogen bonds, whereas the motility assay revealed the difference upon losing the last hydrogen bond of the arm-motif. This mechanistic insight would have not been revealed by simply studying defects in gliding motility and shows the power of the combination of biochemical with biophysical and computational assays. The data suggests a threshold, such that a successively lower affinity between profilin and actin leads first to the loss of force generation (concomitant with an increase in retrograde flow speed) followed by a loss in the capacity to glide on a 2D substrate (Table 3). Further disruption of actin dynamics or the organization of actin filaments would then lead to additional problems such as salivary gland invasion [30, 31, 35].

**Table 3:** Summary of results, including previous work, revealing a possible thresholding phenomenon during sporozoite motility.

*P*. *berghei* parasites	Retrograde flow rate	Force production	Sporozoite motility^1^	Actin dynamics^2^	Actin-profilin interaction
WT cyto D (200 nM)^3^	+	+/-	+/-	+/-	+++
WT cyto D (50 nM)^3^	++	+	++	+	+++
**WT**	**++**	**+++**	**+++**	**+++**	**+++**
WT jas (50 nM)^3^	++++	+	++	++	+++
*tlp(-)*^3^	++++	+	+++	++	+++
*Pf* Pfn	++	+++	++	+++	+++
QNQ Pfn	+++	+	++	++	++
AAA Pfn	+++	+	+	+	+
*coronin(-)*^4^	n.a.	n.a.	+/-	+/-	+++

Light grey background corresponds to robust motility in 2D and 3D, while dark grey highlights robust motility in 3D but not 2D environments.

^1^Sporozoite motility *in vitro* is plotted as a semi-quantitative parameter taking into account the percentage of motile sporozoites and their speed.

^2^Hypothetical as actin filaments and their dynamics have not been directly observed.

^3^taken from [20, 46]

^4^taken from [30]–note that lack of the actin filament bundling coronin likely leads to misorientation of actin filaments; n.a.: not applicable as these parasites could not be tested with optical traps due to their low level of substrate adhesion.

### How is force generated for motility?

**Fig 7:**
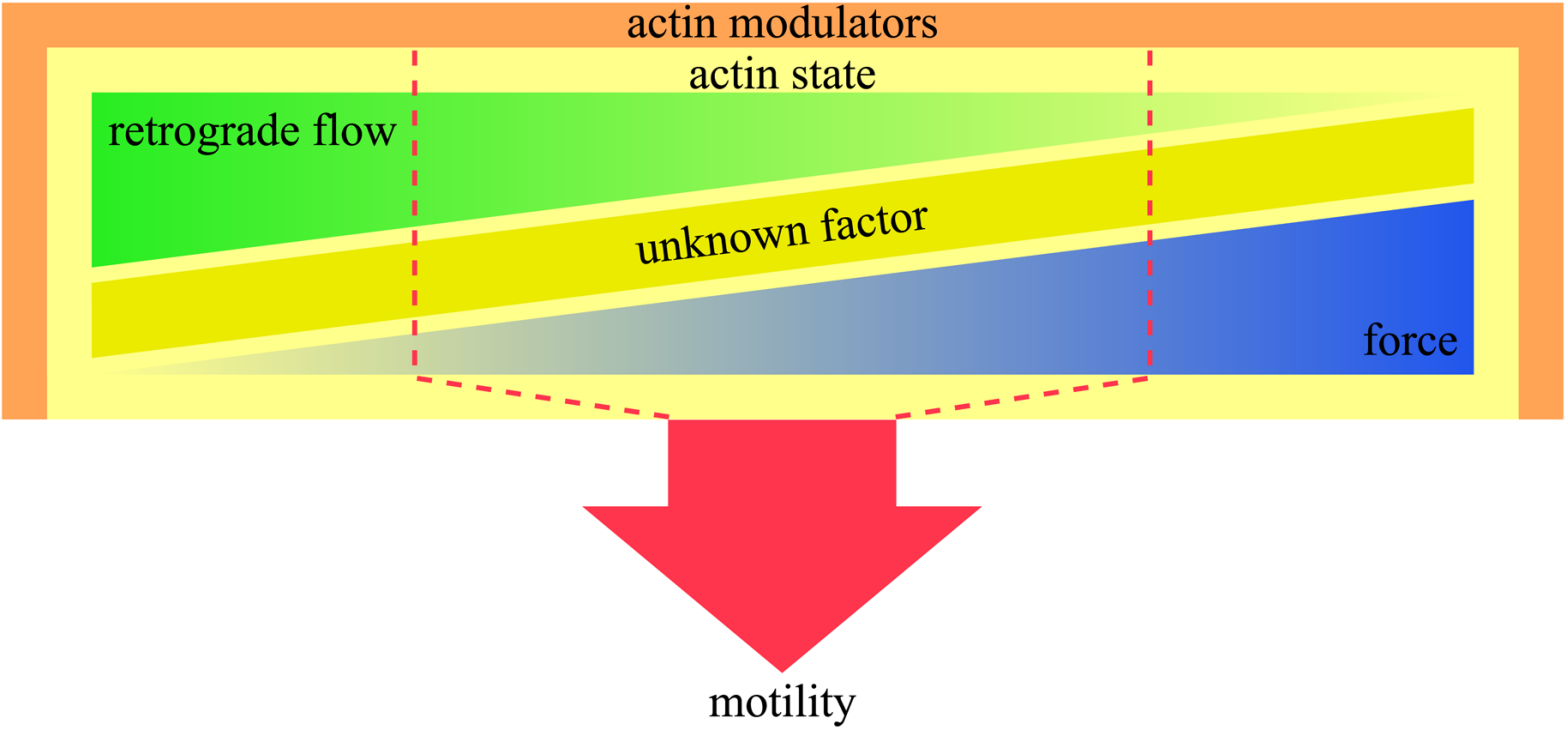
Schematic cartoon summarizing the complex interplay of factors leading to sporozoite motility. Actin modulators such as profilin (this study) or coronin [30] influence the state of actin by either promoting or preventing filament formation, filament crosslinking and filament orientation. The actin state influences retrograde flow and force generation as revealed by the use of inhibitors or mutants. Motility is the outcome of a non-trivial interplay between retrograde flow and force as these are not perfectly inversely correlated. Hence we stipulate the existence of another unknown factor that influences the actin state to produce optimal motility. The system is robust within a certain range (red dashed line), while unbalancing one or several of these factors leads to aberrant motility.

We had previously hypothesized that integration of retrograde flow and force production produces motility [20]. However, the near-normal motile behavior of the QNQ mutant could not have been predicted through these two parameters alone. This leads us to postulate that an as yet unknown factor plays a role in producing sporozoite motility (Fig 7). This factor could be a molecule, a molecular complex or the dynamic arrangement of proteins in complexes. Considering the complexity of the interplay between many factors to generate motility, we favor the latter hypothesis.

Interesting in this respect and for the future direction of research into parasite motility that goes beyond the identification of molecular players are two recent studies on *T*. *gondii* tachyzoites. The first study adapted our laser tweezer approach to *T*. *gondii* tachyzoites [47]. This revealed surprising differences compared to sporozoites. Most strikingly, retrograde flow in tachyzoites occurred after a delay of approximately one minute, while it was instantaneous in sporozoites. Also, directed force production only occurred after this initial phase in tachyzoites. This clearly suggests different kinetics to assemble a directional motor between the two parasites. A second study investigating the essential role of many of the key proteins involved in motility showed that parasites can still be motile, albeit at a much reduced rate, in the absence of core motility proteins, including actin [48]. This showed that even in the absence of actin, retrograde flow still moved beads towards the rear of the tachyzoite. Yet, retrograde flow occurred in a manner of minutes and not seconds as observed with *P*. *berghei* sporozoites. This suggests a much more complex picture and the existence of important differences between apicomplexan parasites that are currently not fully appreciated.

### Actin dynamics influences force generation in sporozoites

Sporozoite motility relies on a complex interaction of the formation and turnover of adhesion sites as well as the generation of force [19–21, 49]. It is currently not clear how this complex interplay is orchestrated. Investigating motility thus relies on the determination of many different factors, such as the percentage of motile sporozoites, and their average and instantaneous speeds (Fig 5, Table 3).

Previously, we have shown that low concentrations of jasplakinolide, which likely leads to longer or less dynamic actin filaments, cause higher retrograde flow speed but lower force [20] and less motility ([46], S1B Fig, Table 3). Cytochalasin D, which in contrast disrupts actin filaments, led to lower force and less motility, yet had only mild effects on retrograde flow ([20], S1B Fig, Table 3). Thus, longer and more stable filaments would lead to a higher retrograde flow, which is the case for both of our mutants (Fig 6D). Both longer filaments and disruption of filaments would lead to lower forces, which again is the case for both mutants (Fig 6B). Thus, the length of filaments and their dynamics do not scale linearly with the loss of force. Rather, maximum force generation likely requires optimized filament length, dynamics, and orientation, which are achieved through the interplay of several proteins *in vivo* [18, 20, 30, 50].

Although both mutants have weakened actin-binding affinity, the QNQ mutant seems to be above a threshold required for gliding. It seems likely that the AAA mutant releases actin monomers faster, so that *in vivo*, other monomer-binding proteins have the chance to bind actin. These could be for example the ADFs, which promote filament formation by catalyzing nucleotide exchange [28, 51]. This also shows that sporozoite motility *in vivo* is robust enough to tolerate small changes in binding affinities of individual proteins, and that our *in vitro* assays are sensitive enough to now understand the fine-tuning of this incredibly efficient and fast machinery.

### Concluding remarks

In summary, we present the first study using site-directed mutagenesis of a constitutively expressed protein involved in malaria parasite motility that uncovers a new type of interaction between actin and profilin. We provide conclusive evidence from a combination of biochemical, biophysical, computational and molecular genetic studies that this actin-profilin interface is important for *Plasmodium* sporozoite migration. We suggest that sporozoite entry into salivary glands is the key constraint for the evolution of the *Plasmodium* motility machinery.

## Methods

### Ethics statement

All animal experiments were performed according to the German Animal Welfare Act (Tierschutzgesetz) and were approved by the responsible German authorities (Regierungspräsidium Karlsruhe, numbers G-3/11, G-134/14 and G-310/14).

### Animal work

Generation of parasite lines and infections of mosquitoes was performed using female NMRI mice (Janvier). Monitoring of parasite prepatency and blood stage growth rates was performed using female C57BL/6 mice (Charles River).

### Recombinant protein production and biochemical work

A mutant *Pf Pfn* construct in which the sequence _64_EDE_66_ was replaced by QNQ was obtained by gene synthesis from Mr. Gene and sub cloned into pETM-11 using NcoI and XhoI restriction sites. This was used as a backbone to generate the AAA mutant using site-directed mutagenesis with the QuikChange Lightning mutagenesis kit (Agilent Technologies) with primer pair 12. Wild type [33] and mutant *Pf* Pfns were expressed and purified [37] and the correct folding of the mutants verified using synchrotron CD spectroscopy, as described before [40] **(**S7 Fig**)**. Pig (*Sus scrofa*) skeletal muscle α-actin was purified as before [37, 52]. *Pf* actin 1 was expressed in *Spodoptera frugiperda Sf*21 cells (Invitrogen) as described earlier [37]. After expression the cells were harvested and suspended in lysis buffer (10 mM HEPES pH 7.5, 250 mM NaCl, 5 mM CaCl_2_, 15 mM imidazole, 1 mM ATP and 7 mM β-mercaptoethanol). Cells were lysed by sonication on ice, and the lysate was clarified by a 1-h centrifugation at 30600 g. The supernatant was mixed with 1 ml of HisPur Ni-NTA resin (Thermo Fisher Scientific), incubated for 1 h at 4°C, and the resin was washed extensively with lysis buffer 1 with 3 mM β-mercaptoethanol, the same buffer with 500 mM NaCl and 25 mM imidazole, and with G-buffer (10 mM HEPES pH 7.5, 0.2 mM CaCl_2_, 0.5 mM ATP and 3 mM β-mercaptoethanol). Finally, the protein was eluted with G-buffer containing 300 mM imidazole and imidazole removed using a PD-10 desalting column (GE Healthcare). The His-tag was cleaved by a 1 h incubation at 22°C with TEV protease, after which the protein was passed through a Ni-NTA column and supplemented with 300 mM ammonium acetate. Finally, the protein was concentrated and applied to a Superdex 200 10/300 GL column (GE Healthcare) in GF-buffer (G-buffer containing 300 mM ammonium acetate and 0.5 mM TCEP-HCl instead of β-mercaptoethanol) for final purification.

The effects of wild type and mutant profilins on α-actin polymerization kinetics were measured using fluorescence spectroscopy. 4 μM actin, of which 5% pyrene-labelled, in 10 mM Tris-HCl, pH 7.5, 0.5 mM ATP, 0.2 mM CaCl_2_, 1 mM DTT was induced to polymerize by the addition of polymerizing buffer to final concentrations of 0.1 M KCl, 0.1 mM MgCl_2_, 5 mM Tris-HCl, pH 7.5, 0.2 mM ATP in the presence of 0–20 μM wild-type *Pf* Pfn or the QNQ and AAA mutants. Polymerization was followed for 1 h by measuring the increase in fluorescence signal upon incorporation of pyrene-labelled actin into growing filaments using a Tecan M1000 Pro plate reader at 25°C with excitation and emission wavelengths of 365 and 407 nm, respectively.

The effects of *Pf* and mutant profilins on *Pf* actin 1 polymerization were studied using fluorescence spectroscopy as in the case of α-actin but using approximately 1 and 2% of pyrene-labelled *Pf* actin 1 in the case of the wild-type *Pf* profilin concentration series and *Pf*, QNQ and AAA profilin comparison experiments, respectively. Polymerization of 4 μM *Pf* actin 1 alone and in the presence of 1–64 μM *Pf* profilin or 16 μM *Pf*, QNQ or AAA profilin (in duplicates) was induced by adding polymerizing buffer to final concentrations of 50 mM KCl, 4 mM MgCl_2_, and 1 mM EGTA. Polymerization was followed for 2 h using the parameters described above.

All polymerization curves were set to start from zero fluorescence intensity, and the initial polymerization rates were determined as the slopes of linear fits to the polymerization data from 300 to 600 s for α-actin and 600 to 1200 s for *Pf* actin 1. The relative initial polymerization rates were obtained by dividing the initial polymerization rate values with the initial polymerization rate of *Pf* actin 1 alone.

For co-sedimentation experiments 100 μl of each *Pf* actin 1 polymerization sample were recovered from the 96-well plate. Samples were centrifuged 1 h at 25°C and 100000 rpm, using a TLA-100 rotor (Beckman Coulter), and the resulting supernatants and pellets were separated. The supernatants were mixed with 25 μl of 5x SDS-PAGE sample buffer (250 mM Tris pH 6.8, 10% SDS, 50% glycerol, 0.02% bromophenol blue, 1.43 M β-mercaptoethanol), and the pellets were resuspended in 125 μl of PBS pH 7.4 supplemented with 1x SDS-PAGE sample buffer. Samples were incubated 5 min at 95°C and 10 μl of each sample was analyzed on 4–20% SDS-PAGE gels. The protein bands were visualized with PageBlue stain (Thermo Fisher Scientific). Gels were imaged using the ChemiDoc XR S+ system and protein band intensities were determined with the Image Lab 3.0 software (both from Bio-Rad). For each supernatant and pellet pair, the total intensity of *Pf* actin 1 was set to 100% and relative amounts of actin 1 in supernatants and pellets were presented as percentages of that.

To study the *Pf* actin 1-profilin interaction on native PAGE, samples containing 5.6 μM *Pf* actin 1 alone and together with increasing concentrations (1–256 μM) of *Pf*, QNQ, and AAA profilins were prepared. In addition, samples with 32 μM profilins alone were included. The samples were incubated for 15 min at 22°C and supplemented with native PAGE loading dye (6% glycerol, 50 mM Tris pH 8.0, 0.004% bromophenol blue, final concentrations). 12.5 μl of each sample were analyzed on a 4–20% MiniProtean TGX gel (Bio-Rad) using 25 mM Tris, 200 mM glycine, 0.5 mM ATP, 0.1 mM CaCl_2_ pH 8.3 as the running buffer and constant 150 V for 1.5 h at 4°C. Finally, the gels were stained with PageBlue protein staining solution (Thermo Scientific).

### Small-angle X-ray scattering

For SAXS analysis, wild-type Pf Pfn and α-actin were mixed in a 1:1 molar ratio, incubated on ice for 2 h, concentrated to 500 μl using a centrifugal filter, and gel filtered using a Superdex S200 10/300 GL column, equilibrated in 5 mM Tris-HCl (pH 7.5), 0.2 mM CaCl_2_, 0.2 mM ATP, and 2 mM DTT. The peak fractions containing the complex were pooled and concentrated to 2.5 mg/ml. SAXS data were collected on beamline X33 at EMBL/DESY, Hamburg (Germany) at concentrations 2.5 and 1.25 mg/ml. Analysis of the data was carried out using the ATSAS package [53]. *Ab initio* models were built using DAMMIF [54] and GASBOR [55]. The Pfn-actin complex had an R_g_ value of 2.4 nm and D_max_ of 8 nm, compared to the respective values of 2.5 and 8.1 for BSA and 1.4 and 4.7 for *Pf* Pfn alone. Actin alone in our experience polymerizes during the SAXS measurement and does not give meaningful values for comparison with globular proteins.

### Molecular dynamics simulations

Preparation of structures: The crystal structures of *P*. *falciparum* actin 1 (PDB ID: 4CBU, 1.3 Å resolution) [25] and *P*. *falciparum* profilin (PDB ID: 2JKF, 2.31 Å resolution) [33] were retrieved from the RCSB-PDB database [56]. These structures were aligned to the crystal structure of rabbit alpha skeletal muscle actin co-crystallized with human profilin (PDB ID: 2PAV, 2.31 Å resolution) [57]. The thus obtained *P*. *falciparum* actin-profilin complex was prepared for simulations using the Protein Preparation Wizard module of Schrodinger (version 2016r1). In brief, the complex was preprocessed to assign bond orders, to add missing hydrogen atoms and to add missing side chains. Co-crystallized waters were kept in the complex structure. PROPKA [58] was used to predict the protonation states at pH 7.0 of the titratable residues. Missing residues were modelled using the Prime module in the Schrodinger software. Note that the *P*. *falciparum* and *P*. *berghei* actin only differ in three amino acid residues E3D, D5E and V11I which are distant from the conventional actin-profilin interface.

To model *P*. *berghei* profilin, the *P*. *berghei* profilin sequence was retrieved from the PlasmoDB database [59] and modelled using PRIME software using the *P*. *falciparum* profilin structure (75.9% identical to *P*. *berghei* profilin) as the template structure. The residues at the tip of the arm of *P*. *falciparum* profilin, _64_EDE_66,_ were mutated using Maestro (Schrodinger) to generate the two mutants: _64_AAA_66_ and _64_QNQ_66._

Molecular dynamics simulations: The modelled protein complexes were prepared for all-atom molecular dynamics simulations using the tleap program in the AMBER molecular dynamics package version 14 (http://ambermd.org/) [60]. ATP parameters were taken from the AMBER parameter database (http://research.bmh.manchester.ac.uk/bryce/amber) [61]. GAFF [62] and ff14SB [60] parameters were assigned to the ligand and protein, respectively. Non-bonded interactions were cut off at 8 Å and PME was applied. The systems were solvated using the TIP3P water model [63] in a truncated octahedral box. K^+^ and Cl^-^ ions were added to obtain an ionic strength of 50 mM and the systems were neutralized using Na^+^ counter-ions. A 2-step minimization was performed on each system as follows: 1000 steps of minimization while keeping restraints (force constant 100 kcal/mol Å^2^) on the solute (protein and ligands) (first 500 steps of steepest descent, next 500 steps of conjugate gradient) followed by all-atom minimization (first 1500 steps of steepest descent, next 1000 steps of conjugate gradient). The minimized systems were gradually heated (0 to 298 K in 80 picoseconds) using the canonical ensemble (NVT) at each temperature point. In the next step, the pre-heated systems were equilibrated in an isothermal–isobaric ensemble (NPT) at 298 K. Berendsen temperature coupling and a constant pressure of 1 atm with isotropic molecule-based scaling was used in the equilibration. The SHAKE algorithm [64] was applied to constrain all covalent bonds containing hydrogen atoms and a time step of 2 fs was used. All systems were simulated with periodic boundary conditions in the NPT ensemble for 150 ns. The analysis of the MD trajectories was carried out with the CPPTRAJ module of AMBER 14. VMD (version 1.9.2), Chimera (version 1.10) and Pymol (version 1.8.2.3) were used for visualization.

Binding free energy calculations: The molecular mechanics energies combined with the Poisson Boltzmann or generalized Born and surface area continuum solvation (MM/PBSA and MM/GBSA) energies were used to estimate the actin-profilin binding free energy. The snapshots were retrieved at an interval of 1 ns from the last 50 ns of the MD trajectories (between 100 and 150 ns). Because the current study involves the comparison of similar systems, we did not explicitly calculate entropic contributions to the binding free energy, assuming they are similar in all cases. Therefore, the calculated energies do not correspond to the absolute free energies but can be used to compare similar systems.

### Molecular cloning and parasite generation

All vectors used in this work are based on the b3D+ vector [65]. We modified the vector for homologous recombination in the profilin (PBANKA_0833000) locus on chromosome 8 as follows. The *P*. *berghei* profilin 5’ upstream region (871 bp) was amplified from *P*. *berghei* ANKA WT genomic DNA using primer combination 5 (see S1 Table) and subsequently inserted into b3D+ *via* SacII and NotI digestion and ligation (S2 Fig). The profilin 3’ downstream region (805 bp) was amplified with primer combination 6 and inserted using ClaI and KpnI. For the Pfn-mCh lines, the *P*. *berghei* profilin gene either from gDNA (+i) or cDNA (-i) was fused to mCherry using overlap extension PCR with primer combinations 7 and 8. The fused genes connected through four glycines as a linker were cloned using NotI and BamHI.

The third tagged line Pfn^-i^ // mCh in which the mCherry tag was cleaved to at least 75%, was created with a longer linker (amino acids: AAAASRTSAAAA; this sequence includes the amino acids encoded by the XbaI and SpeI restriction sites). *P*. *berghei* wild type profilin was amplified with primer combination 9 and cloned with NotI and XbaI. The mCherry gene was amplified with primer combination 10 and cloned using SpeI and BamHI. As a control, the Heussler group (Bern University, Switzerland) kindly provided us with a parasite expressing cytoplasmic mCherry under the ef1α promoter [66]. *P*. *falciparum* wild type and mutant (QNQ & AAA) profilins were amplified from the respective pETM-11 vectors used for protein expression with primer combination 11 and cloned into b3D+ using NotI and XbaI.

### Parasite transfection and sporozoite generation

All transfections and generation of clonal parasite lines were performed as previously described [67]. All vectors were linearized with SacII and KpnI prior to transfection. A PCR to probe correct integration was performed after limiting dilution cloning (S2 Fig). *Anopheles stephensi* mosquitoes were infected with clonal lines as follows: Infected mouse blood was injected intraperitoneally (IP) into a naïve NMRI mouse. At a parasitemia of >1% the blood was harvested and 10–20 million parasites were injected IP into two or three naïve mice. Three to four days later, mice were anesthetized and positioned on top of a mosquito cage so as to allow mosquitoes to feed. Mosquitoes were analyzed for oocyst numbers on day 12 and for midgut and salivary gland sporozoites on days 17–23.

### Assessment of parasite function across the life cycle

We determined parasite growth rates by injecting 100 or 5000 infected red blood cells into each of four C57BL/6 mice respectively. Parasitemia was monitored daily from day 3 on. We calculated parasite growth rate as explained before [68, 69]. Oocyst numbers were determined by extracting midguts of infected mosquitoes on day 12 after infection. Midguts were stained for 20 min using 0.1% mercurochrome solution [70]. Stained oocysts were counted using a 10x objective in at least 50 infected midguts.

Imaging was performed using an inverted Axiovert 200 M microscope (Zeiss, Göttingen). Blood stages were imaged by applying a drop of blood from the tail vein of an infected mouse, adding 1 μg / ml Hoechst 33342 DNA dye and diluting it with PBS. The mixture was covered with a cover slip and imaged with differential interference contrast (DIC) and fluorescence microscopy using the same exposure times and objective lens (63x, N.A. 1.4).

Liver stages were fixed with 4% paraformaldehyde solution, permeabilized using 0.5% of Triton-X 100 and stained with α-HSP70 antibody [71] and α-mCherry antibody (ab183628, abcam).

### Quantification of sporozoite and ookinete motility

Ookinete cultures were prepared as previously described [72]. Imaging of motile ookinetes was performed by applying a drop of the culture onto a glass slide and covering it with a cover slip. DIC images were acquired at 0.05 Hz for 15 min. Salivary glands of infected mosquitoes were isolated between days 17 and 25 after infection. They were kept in RPMI (supplemented with 50 000 units / l penicillin and 50 mg /l streptomycin) containing 3% bovine serum albumin (BSA, Roth Ltd) and transferred to a 96 well plate (Nunc MicroWell 96 well optical bottom plates, Sigma) for imaging. The plate was centrifuged for 5 min at 500 g to settle the sporozoites. DIC images were acquired at 0.33 Hz for 5 min. Sporozoite and ookinete speeds were analyzed using the ImageJ plug-in ‘Manual tracking’ [73].

### Retrograde flow experiments

The retrograde flow experiments were performed on the self-built laser trap setup described in [20]. In brief, beads (PC-S-2.0, streptavidin-polystyrene microparticles 1.5–1.9 μm, 1% w/v, Kisker) were held with minimal laser power by a stationary laser trap. Consequently the stage and thereby the self-built open flow cell with the gliding sporozoites in it, were moved towards an optically trapped bead. The bead was then positioned onto the front end of the sporozoite. When sporozoite and bead made contact, the sporozoite pulled the bead out of the focus of the laser and translocated the bead to rear of the cell. This was imaged with a frame rate of 100 images per second. The speeds of the transported beads were tracked using MATLAB routines.

### Force measurements

The force measurement experiments were performed as described in detail in [20]. Beads were captured in the center of the trap—this time with defined forces (70 pN, 130 pN and 190 pN)—were brought in close proximity with the sporozoite until they touched the beads. Sporozoites were challenged to displace the bead from the focus of the trap.

## Supporting information

S1 Fig The actin-myosin motor in sporozoites and the role of actin dynamics in motility. (A) Schematic of the minimal motor machinery depicting myosin anchored in the inner membrane complex (IMC) and underlying subpellicular network (SPN). An actin filament (green) is shown to be linked to plasma membrane (PM) spanning TRAP family adhesins (blue). (B) The wild type (Wt) profilin contributes to highly dynamic actin filaments by sequestering actin monomers. This ensures optimal sporozoite gliding motility. Cytochalasin binds to the barbed end of actin filaments and blocks addition of monomers leading overall to shorter filaments as filaments still shrink from their pointed ends. Jasplakinolide binds to and stabilizes actin filaments thus decreasing the off-rate of actin from filaments and also shifting to lower actin dynamics.
(TIF)

S2 Fig Generation of *P*. *berghei* parasite lines expressing wild type and mutated *P*. *falciparum* profilin or *P*. *berghei* profilin-mCherry fusion proteins. (A) Wild type profilin locus (i) and transgenic loci after replacement with *P*. *berghei* profilin-mCherry (ii) or wild type and mutant *P*. *falciparum* profilin (iii). (B) PCR analysis of the obtained mCherry-tagged clones without or with introns. Note the increase in size of fragments 1b and 3 in Pfn^+i^ mCh of 685 bases (introns) compared with the Pfn^-i^ mCh clone. Expected fragment sizes are indicated below the gels in B and C. (C) PCR analysis of the obtained *P*. *berghei* clones expressing *P*. *falciparum* wild type or mutant profilins.
(TIF)

S3 Fig Expression analysis of parasite lines. (A) Western blot showing profilin-mCherry fusion proteins as detected with an anti-mCherry antibody in blood stage schizont lysates for the indicated parasite lines. Loading control: anti-HSP70; * denotes an unspecific or degradation product. (B) Profilin is expressed throughout the parasite life cycle within the cytoplasm and nucleus. Note the enrichment of profilin-mCherry in the ookinete nucleus. Scale bar: 5 μm. (C, D) Relative (C) and total (D) intensity of mCherry tagged profilin in ring stages and trophozoites of the lines with (blue) and without (black) introns plotted over their cell volume. This representation shows that small cells contain the highest concentration of profilin-mCherry suggesting that profilin is strongly expressed in early ring stages. Note that parasites without introns in the profilin gene appear to be larger (increased number of black dots at higher cell volumes).
(TIF)

S4 Fig Small angle X-ray scattering analysis of the actin-profilin complex. (A) Cartoon representation of the *Pf* Pfn-α-actin complex based on superposition of canonical Pfn-actin complexes. (B) DAMMIF and (C) GASBOR *ab initio* models show a similar shape compared to the canonical Pfn-actin complex. (D) Fit of the DAMMIF model (green) to the SAXS data (black). (E) Fit of the GASBOR model (yellow) to the SAXS data (black). The χ^2^ values are 0.92 and 3.5, respectively.
(TIF)

S5 Fig Effect of *Pf* Pfn binding on pyrene-actin fluorescence. Binding of *Pf* Pfn increases the fluorescence intensity of pyrene-labelled actin for both *Pf* actin 1 (A) and skeletal muscle α-actin (B). For the QNQ (C) and AAA (D) mutants, such an increase with α-actin is not visible at the concentrations used.
(TIF)

S6 Fig Analysis of the canonical actin profilin interaction site. Representative conformations of the three actin-profilin complexes from MD simulations show the interacting residues. The proteins are shown in cartoon representation with actin in gold and profilin in grey. H-bonding residues are shown as sticks. Profilin residues are colored based on the region: red (arm), green (region near to arm) and blue (distant region from arm). Actin residues are shown in white with N atoms blue and O atoms red. H-bonds are shown by magenta dash lines. Residues that do not make H-bonds in one complex but make H-bonds in other structures are labelled in grey (right panel).
(TIF)

S7 Fig Purification of recombinant proteins and effects of *Pf* and mutant profilins on *Pf* actin 1 sedimentation. (A) Size-exclusion chromatogram of the purified wild-type (green) and both mutant (blue) profilins. The purity of the samples is shown on a Coomassie-stained SDS-PAGE gel in the inset. The samples are: 1) wild type *Pf* Pfn, 2) the QNQ mutant, 3) the AAA mutant, 4) molecular-weight standards. The molecular weights of the relevant standards in kDa are shown on the right. (B) The folding of the mutant profilins (blue curves) was shown to be identical to that of the wild-type protein (green) using CD spectroscopy. (C) Sedimentation of 4 μM *Pf* actin 1 alone and in the presence of 1–64 μM *Pf* profilin. (D) Sedimentation of 4 μM *Pf* actin 1 alone and in the presence of 16 μM *Pf*, QNQ and AAA profilins. Samples were analyzed on 4–20% SDS-PAGE gels and protein bands were visualized with PageBlue stain (Thermo Fisher Scientific). S denotes supernatant and P pellet. Quantification from duplicate gels is presented in Fig 3B.
(TIF)

S1 Table Primer pairs with sequences used for cloning and verification of integration. Respective restriction enzyme motifs (lowercase) and binding site descriptions are indicated. Start codons are coloured green and stop codons red. Blue shows the glycine and orange the alanine linker.
(PDF)

S1 Movie 360° rotation of the models of the *P*. *falciparum* profilin-actin I complex from molecular dynamics simulations corresponding to Fig 2A. The first rotation shows actin in gold and profilin in grey; the actin-profilin complex is shown with different contact residues highlighted in red (tip of hairpin), green (arm-neighbor interactions) and blue (classic actin-profilin interface). The second rotation shows surface charges in actin that are highlighted in blue (basic) and red (acidic). Note the tip of the arm-like β-hairpin (acidic amino acids in red) contacting a basic region of actin subdomain 3.
(MP4)

S2 Movie The 150 ns molecular dynamics simulations for the indicated *Plasmodium* actin-profilin complexes corresponding to Fig 2C and 2D. Data analysis for Fig 2 was performed on the last 50 ns of the simulations to exclude artifacts from the initial stabilization. Note how the arm motif in the AAA Pfn complex moves away from actin, as there are no hydrogen bonds formed with the tip of the arm motif.
(MP4)
