# Supplementary figures and images for: A unique profilin-actin interface is important for malaria parasite motility
Source: PLoS Pathog. 2017 May 26;13(5):e1006412. doi: 10.1371/journal.ppat.1006412 (PMC5464670; doi:10.1371/journal.ppat.1006412)

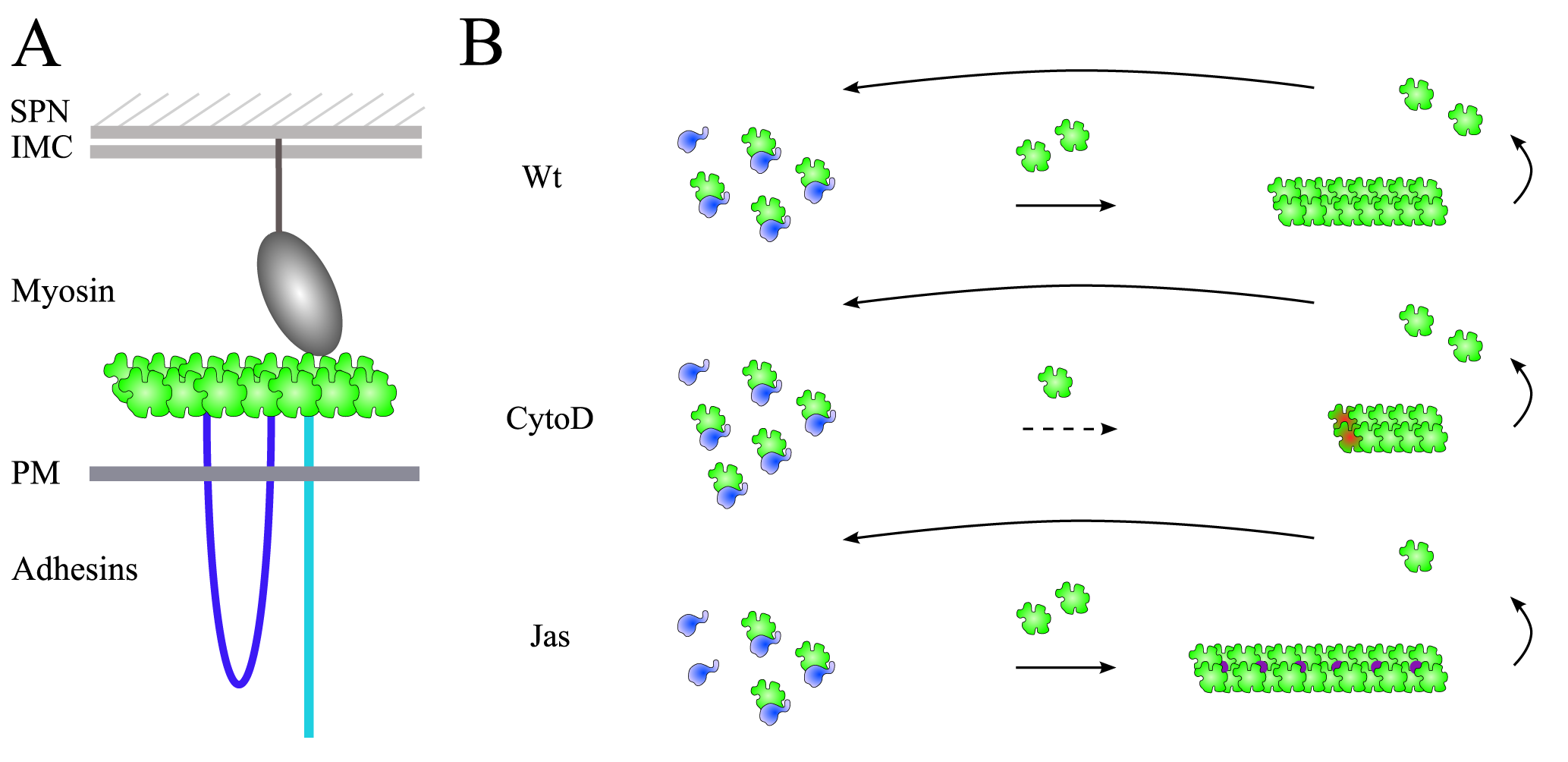

Supplement: S1 Fig — (A) Schematic of the minimal motor machinery depicting myosin anchored in the inner membrane complex (IMC) and underlying subpellicular network (SPN). An actin filament (green) is shown to be linked to plasma membrane (PM) spanning TRAP family adhesins (blue). (B) The wild type (Wt) profilin contributes to highly dynamic actin filaments by sequestering actin monomers. This ensures optimal sporozoite gliding motility. Cytochalasin binds to the barbed end of actin filaments and blocks addition of monomers leading overall to shorter filaments as filaments still shrink from their pointed ends. Jasplakinolide binds to and stabilizes actin filaments thus decreasing the off-rate of actin from filaments and also shifting to lower actin dynamics. (TIF) [file ppat.1006412.s001.tif]

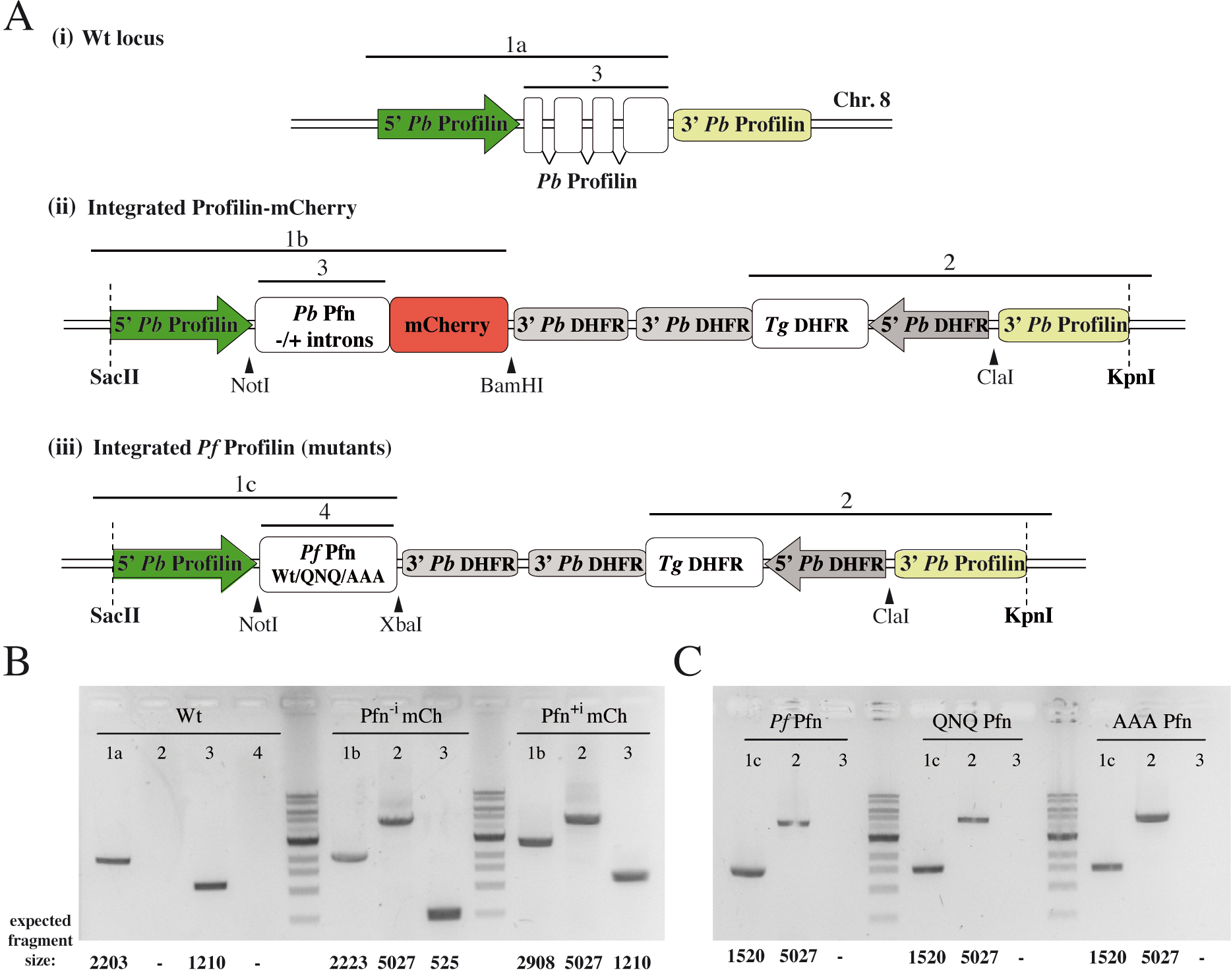

Supplement: S2 Fig — (A) Wild type profilin locus (i) and transgenic loci after replacement with P. berghei profilin-mCherry (ii) or wild type and mutant P. falciparum profilin (iii). (B) PCR analysis of the obtained mCherry-tagged clones without or with introns. Note the increase in size of fragments 1b and 3 in Pfn+i mCh of 685 bases (introns) compared with the Pfn-i mCh clone. Expected fragment sizes are indicated below the gels in B and C. (C) PCR analysis of the obtained P. berghei clones expressing P. falciparum wild type or mutant profilins. (TIF) [file ppat.1006412.s002.tif]

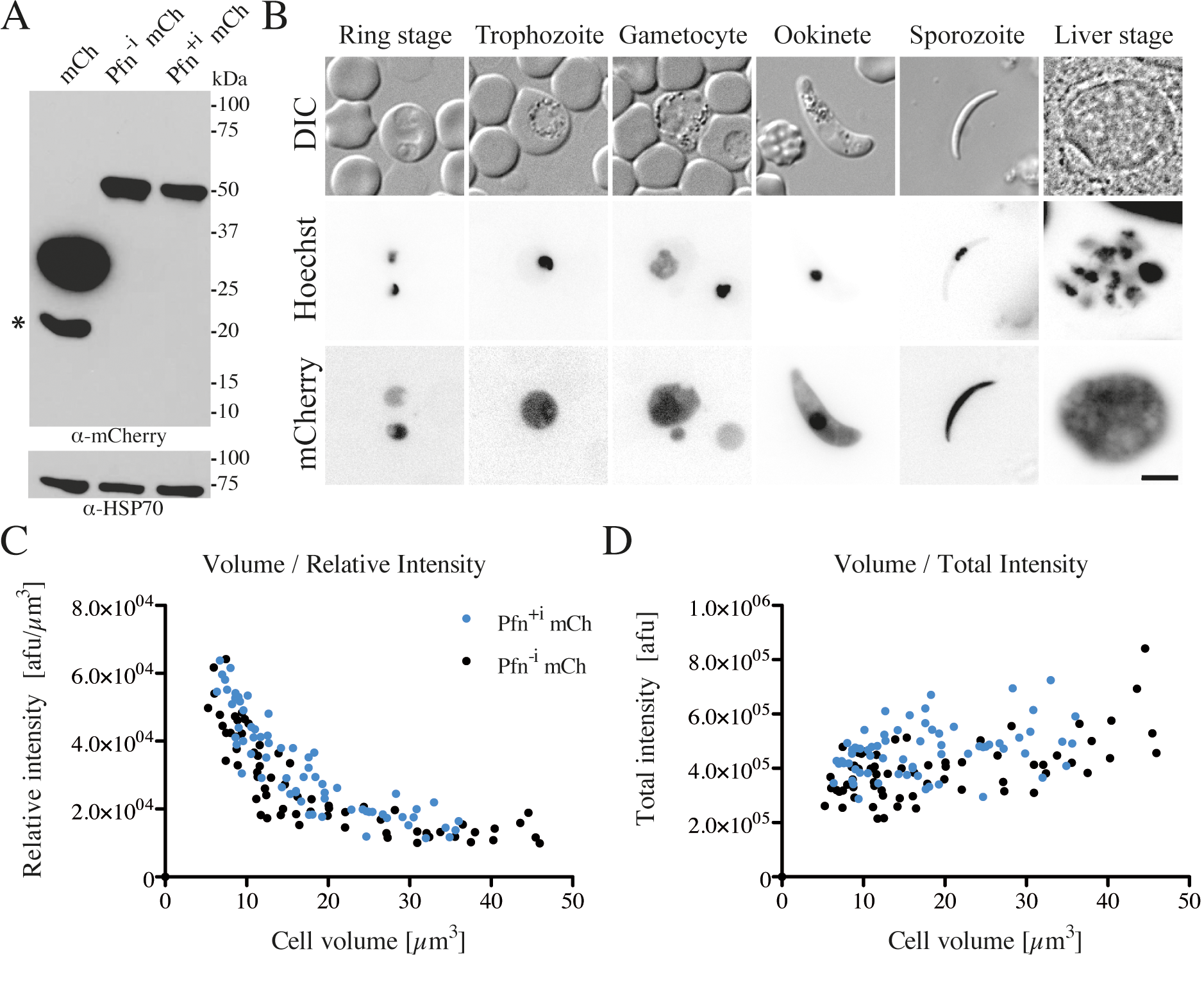

Supplement: S3 Fig — (A) Western blot showing profilin-mCherry fusion proteins as detected with an anti-mCherry antibody in blood stage schizont lysates for the indicated parasite lines. Loading control: anti-HSP70; * denotes an unspecific or degradation product. (B) Profilin is expressed throughout the parasite life cycle within the cytoplasm and nucleus. Note the enrichment of profilin-mCherry in the ookinete nucleus. Scale bar: 5 μm. (C, D) Relative (C) and total (D) intensity of mCherry tagged profilin in ring stages and trophozoites of the lines with (blue) and without (black) introns plotted over their cell volume. This representation shows that small cells contain the highest concentration of profilin-mCherry suggesting that profilin is strongly expressed in early ring stages. Note that parasites without introns in the profilin gene appear to be larger (increased number of black dots at higher cell volumes). (TIF) [file ppat.1006412.s003.tif]

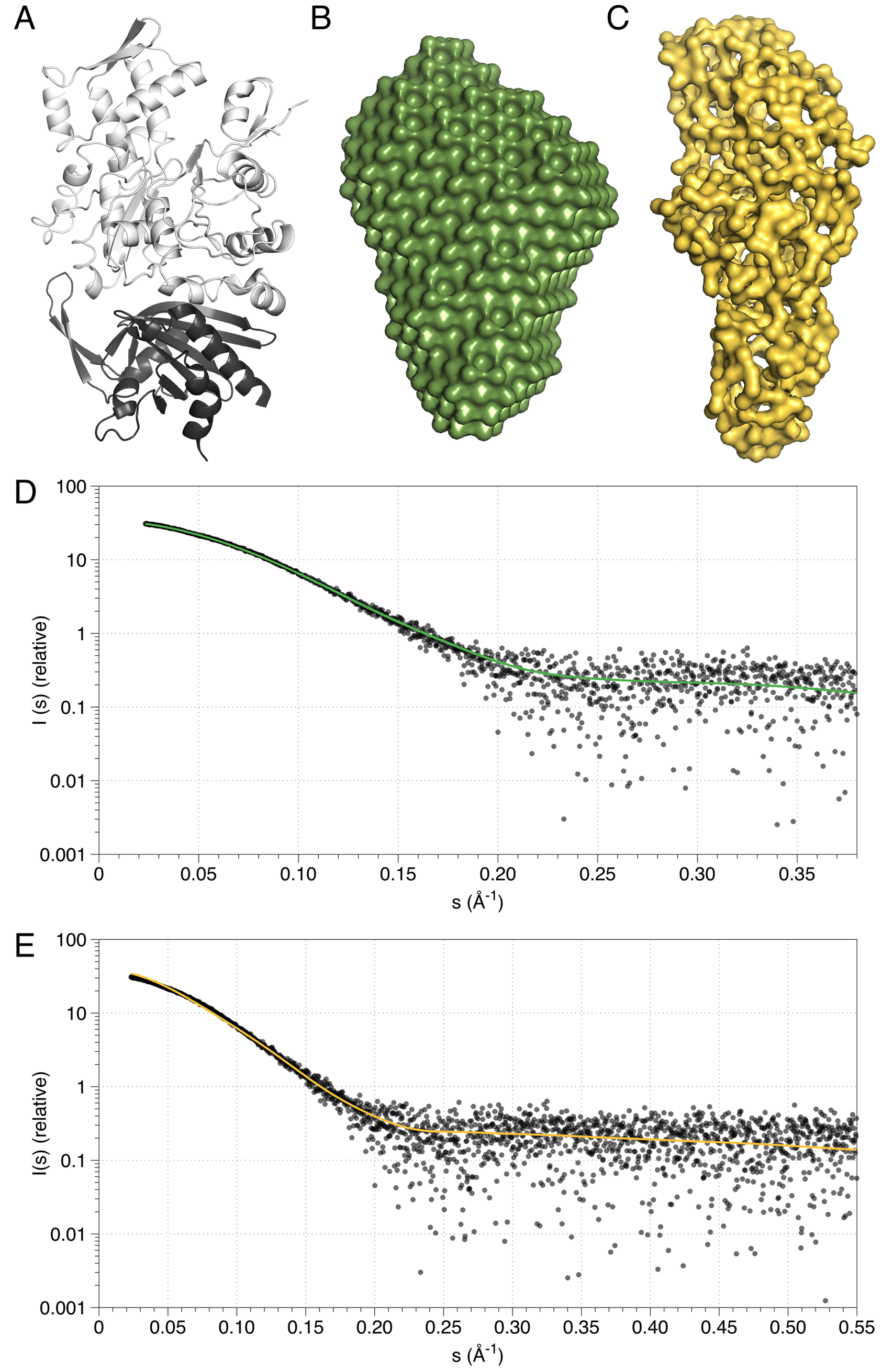

Supplement: S4 Fig — (A) Cartoon representation of the Pf Pfn-α-actin complex based on superposition of canonical Pfn-actin complexes. (B) DAMMIF and (C) GASBOR ab initio models show a similar shape compared to the canonical Pfn-actin complex. (D) Fit of the DAMMIF model (green) to the SAXS data (black). (E) Fit of the GASBOR model (yellow) to the SAXS data (black). The χ2 values are 0.92 and 3.5, respectively. (TIF) [file ppat.1006412.s004.tif]

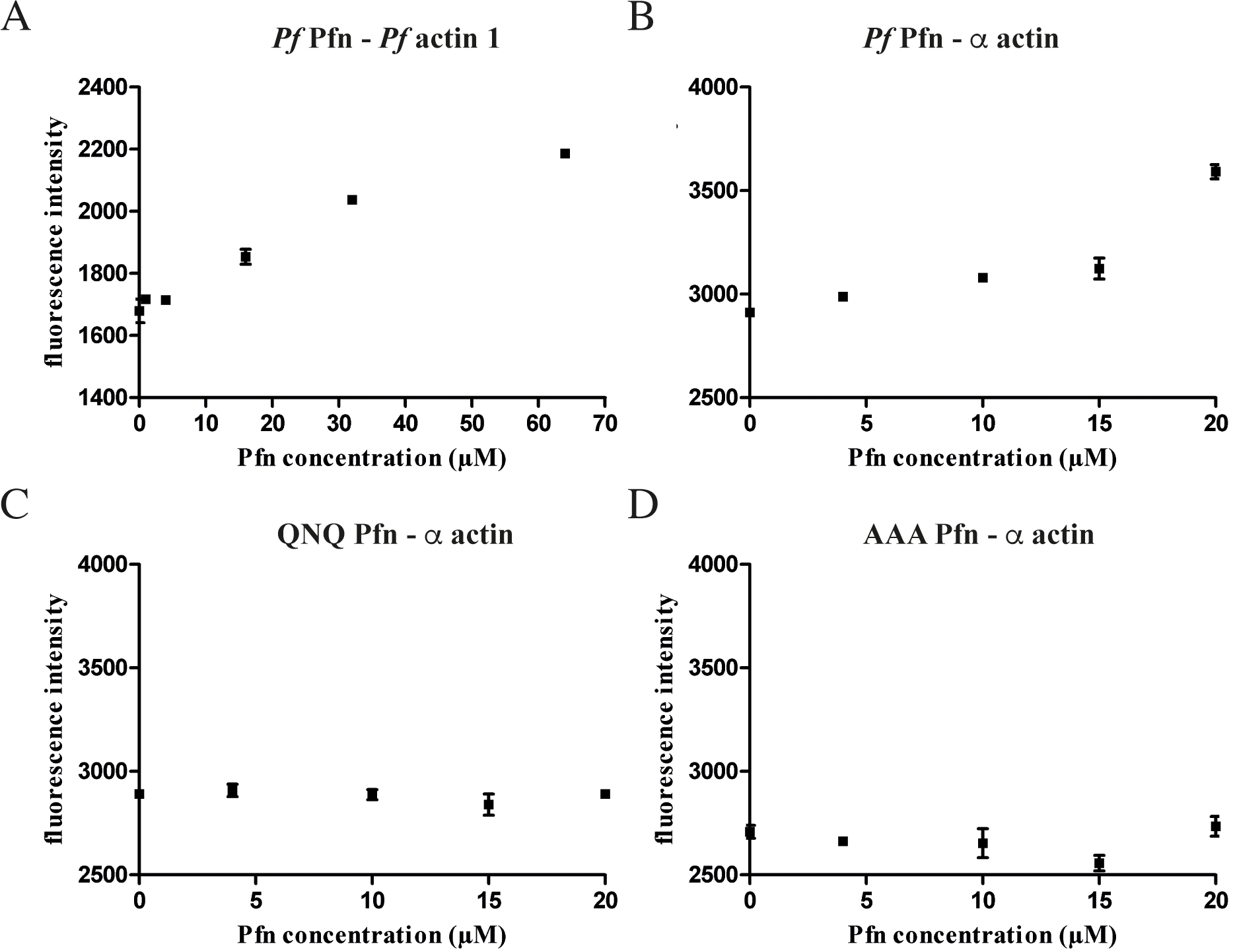

Supplement: S5 Fig — Binding of Pf Pfn increases the fluorescence intensity of pyrene-labelled actin for both Pf actin 1 (A) and skeletal muscle α-actin (B). For the QNQ (C) and AAA (D) mutants, such an increase with α-actin is not visible at the concentrations used. (TIF) [file ppat.1006412.s005.tif]

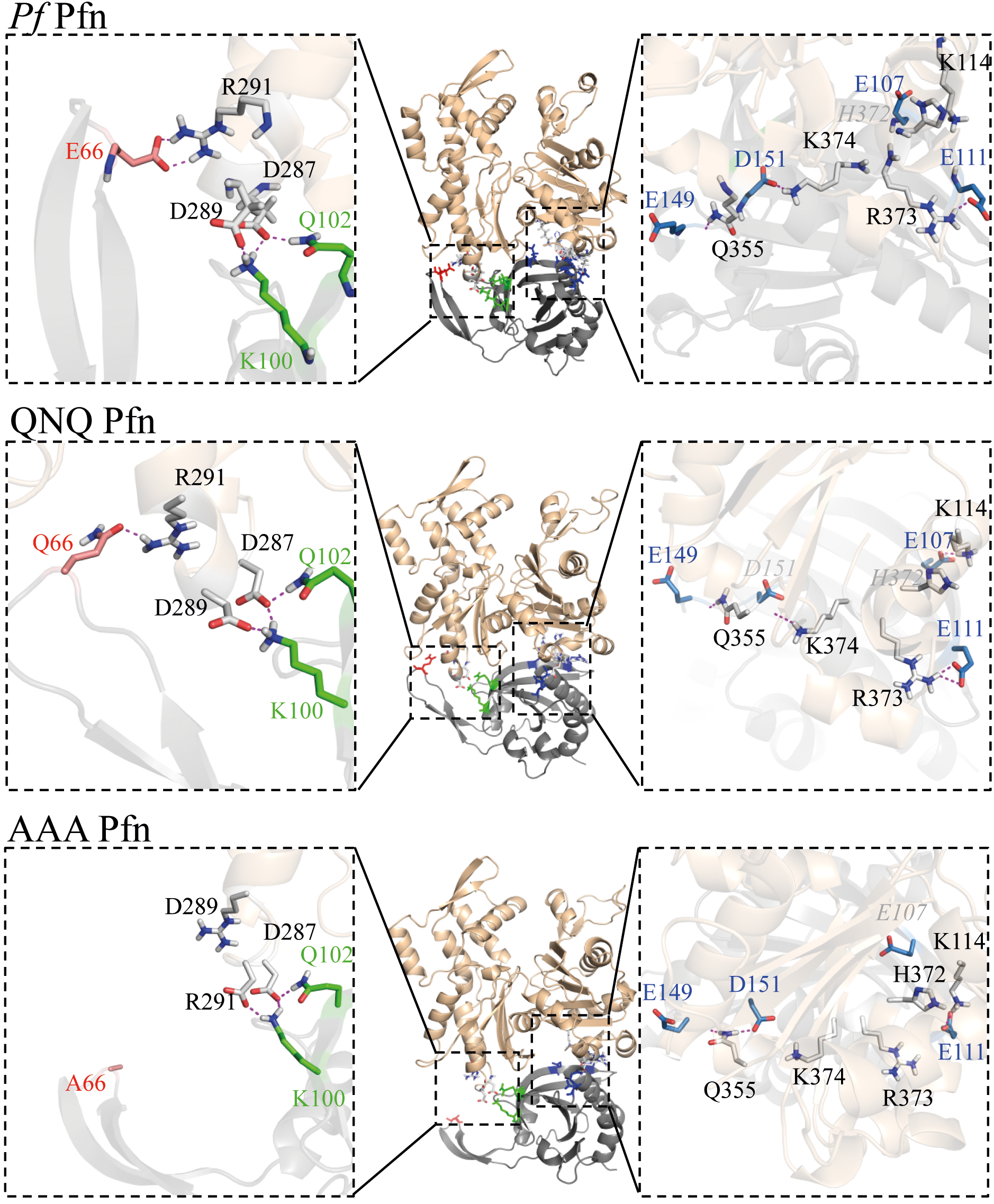

Supplement: S6 Fig — Representative conformations of the three actin-profilin complexes from MD simulations show the interacting residues. The proteins are shown in cartoon representation with actin in gold and profilin in grey. H-bonding residues are shown as sticks. Profilin residues are colored based on the region: red (arm), green (region near to arm) and blue (distant region from arm). Actin residues are shown in white with N atoms blue and O atoms red. H-bonds are shown by magenta dash lines. Residues that do not make H-bonds in one complex but make H-bonds in other structures are labelled in grey (right panel). (TIF) [file ppat.1006412.s006.tif]

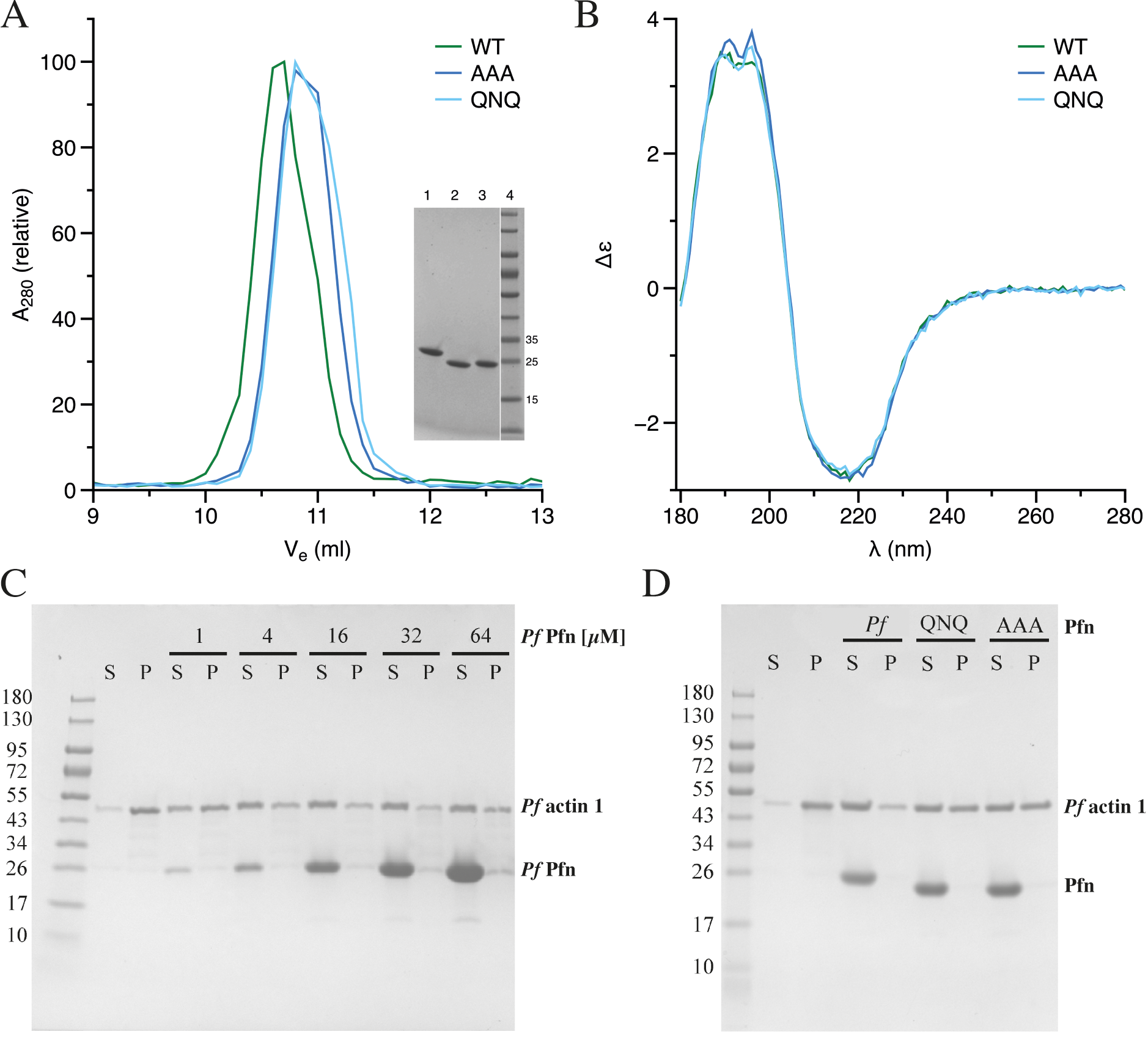

Supplement: S7 Fig — (A) Size-exclusion chromatogram of the purified wild-type (green) and both mutant (blue) profilins. The purity of the samples is shown on a Coomassie-stained SDS-PAGE gel in the inset. The samples are: 1) wild type Pf Pfn, 2) the QNQ mutant, 3) the AAA mutant, 4) molecular-weight standards. The molecular weights of the relevant standards in kDa are shown on the right. (B) The folding of the mutant profilins (blue curves) was shown to be identical to that of the wild-type protein (green) using CD spectroscopy. (C) Sedimentation of 4 μM Pf actin 1 alone and in the presence of 1–64 μM Pf profilin. (D) Sedimentation of 4 μM Pf actin 1 alone and in the presence of 16 μM Pf, QNQ and AAA profilins. Samples were analyzed on 4–20% SDS-PAGE gels and protein bands were visualized with PageBlue stain (Thermo Fisher Scientific). S denotes supernatant and P pellet. Quantification from duplicate gels is presented in Fig 3B. (TIF) [file ppat.1006412.s007.tif]
