# Supplementary material for: A unique profilin-actin interface is important for malaria parasite motility
Source: PLoS Pathog. 2017 May 26;13(5):e1006412. doi: 10.1371/journal.ppat.1006412 (PMC5464670; doi:10.1371/journal.ppat.1006412)
Supplement: S1 Table — Respective restriction enzyme motifs (lowercase) and binding site descriptions are indicated. Start codons are coloured green and stop codons red. Blue shows the glycine and orange the alanine linker. (PDF) [file ppat.1006412.s008.pdf]

Primer	Sequence, Type	Restriction enzyme	Binding site
	Integration Primers		
1 fw	GGTGCACACTCATTTGAATGTG	-	*Pb* Pfn 5' UTR, upstream of homologous region
1a rv	GCtctaga**TTA**TGCGGCACCTGTATCAG	XbaI	*Pb* Pfn end of CDS
1b rv	CGggatcc**TTA**CTTGTACAGCTCGTCCATG	BamHI	mCherry end of CDS
1c rv	GCtctaga**TTA**CTGTGAGCTTTCTGCCAG	XbaI	*Pf* mutant Pfn end of CDS
2 fw	**CTA**GACAGCCATCTCCATCTGG	-	*Tg* DHFR end of CDS
2 rv	CAAGTTCTTTCCTCATGTGTTCATG	-	*Pb* Pfn 3'UTR, downstream of homologous region
3 fw	ATTTgcggccgc**ATG**GAAGAATATTCATGGG	NotI	*Pb* Pfn start of CDS
3 rv	GCtctaga**TTA**TGCGGCACCTGTATCAG	XbaI	*Pb* Pfn end of CDS
4 fw	ATTTgcggccgcAAA**ATG**GCAGAGGAGTATTCTTGG	NotI	*Pf* mutant Pfn start of CDS
4 rv	GCtctaga**TTA**CTGTGAGCTTTCTGCCAG	XbaI	*Pf* mutant Pfn end of CDS
	Cloning primers		
5 fw	TCCccgcggGAGATATTACACATTGCTAC	SacII	*Pb* Pfn 5' UTR fw
5 rv	TAAAgcggccgcCTTTATTATCTTAAAAATTATTTATATAATATGATG	NotI	*Pb* Pfn 5' UTR rv
6 fw	CCatcgatAATAAAGAAAATATTATAAAAATGTG	ClaI	*Pb* Pfn 3' UTR fw
6 rv	GGggtaccCACACATTGGCATTATATAGAAATTGAG	KpnI	*Pb* Pfn 3' UTR rv
7 fw	ATTTgcggccgc**ATG**GAAGAATATTCATGGG	NotI	*Pb* Pfn start of CDS
7 rv	CGCCCTTGCTCAC**GCCGCCGCCGCC**TGCGGCACCTG	-	*Pb* Pfn end of CDS without stop codon
8 fw	CAGGTGCCGCA**GGCGGCGGCGGC**GTGAGCAAGGGCG	-	mCherry start of CDS
8 rv	CGggatcc**TTA**CTTGTACAGCTCGTCCATG	BamHI	mCherry end of CDS
9 fw	ATTTgcggccgc**ATG**GAAGAATATTCATGGG	NotI	*Pb* Pfn start of CDS
9 rv	GCtctaga**TGCTGCTGCTGC**TGCGGCACCTGTATCAG	XbaI	*Pb* Pfn end of CDS without stop codon
10 fw	GCactagt**GCAGCAGCAGCA**GTGAGCAAGGGCGAGGAGGATAACATGG	SpeI	mCherry start of CDS
10 rv	CGggatcc**TTA**CTTGTACAGCTCGTCCATG	BamHI	mCherry end of CDS
11 fw	ATTTgcggccgcAAA**ATG**GCAGAGGAGTATTCTTGG	NotI	*Pf* mutant Pfn start of CDS
11 rv	GCtctaga**TTA**CTGTGAGCTTTCTGCCAG	XbaI	*Pf* mutant Pfn end of CDS
12 fw	ACTATGACATCGAGGTCGCAGCTGCAAACGGGACGAAAACCA	-	Arm region: mutagenesis from QNQ to AAA
12 rv	GTGGTTTTCGTCCCGTTTGCAGCTGCGACCTCGATGTCATAGTCCT	-	Arm region: mutagenesis from QNQ to AAA

restriction sites in lower case, start codons indicated in green, stop codons indicated in red, glycine linker in blue, alanine linker in orange
